# Optimizing automated phase-targeted auditory stimulation protocols for procedural memory consolidation during sleep in a home setting

**DOI:** 10.1093/sleepadvances/zpaf073

**Published:** 2025-10-17

**Authors:** Vanessa Kasties, Nicole Meier, Nora-Hjördis Moser, Renske Sassenburg, Walter Karlen, Maria Laura Ferster, Sara Fattinger, Angelina Maric, Reto Huber

**Affiliations:** Child Development Centre, University Children’s Hospital Zurich, University of Zurich, Lenggstrasse 30, CH-8008 Zurich, Switzerland; Child Development Centre, University Children’s Hospital Zurich, University of Zurich, Lenggstrasse 30, CH-8008 Zurich, Switzerland; Department of Neurology, University Hospital Zurich (USZ), University of Zurich, Zurich, Switzerland; Department of Neurology, University Hospital Zurich (USZ), University of Zurich, Zurich, Switzerland; Institute of Biomedical Engineering, University of Ulm, Ulm, Germany; Child Development Centre, University Children’s Hospital Zurich, University of Zurich, Lenggstrasse 30, CH-8008 Zurich, Switzerland; Child Development Centre, University Children’s Hospital Zurich, University of Zurich, Lenggstrasse 30, CH-8008 Zurich, Switzerland; Department of Neurology, University Hospital Zurich (USZ), University of Zurich, Zurich, Switzerland; Child Development Centre, University Children’s Hospital Zurich, University of Zurich, Lenggstrasse 30, CH-8008 Zurich, Switzerland; Department of Child and Adolescent Psychiatry, Psychiatric Hospital, University of Zurich, Zurich, Switzerland

**Keywords:** slow-wave sleep, auditory stimulation, procedural memory consolidation, mobile application, automated, electroencephalography, K-complex, spindles

## Abstract

Up-phase-targeted auditory stimulation (up-PTAS) during slow-wave sleep has become a valuable tool for modulating slow oscillations and slow oscillation-spindle-coupling in favor of overnight memory retention. Developing effective, automated protocols for translation into more naturalistic and clinical settings is an ongoing challenge, especially because current PTAS protocols and their behavioral effects vary greatly between studies. Our study contributes to ongoing efforts in characterizing parameter choices in PTAS and compares two up-PTAS protocols with systematic variations of the interstimulus intervals (ISIs) and their effect on the consolidation of a finger-tapping sequence using a mobile PTAS device and an app-based behavioral task in a home setting. Participants tolerated both protocols well and showed high adherence to the study procedures. Electrophysiological stimulus responses and learning trajectories in the finger-tapping task replicated lab-based findings. We extend studies suggesting a nonlinear relationship between stimulus number and PTAS effects by showing that applying fewer stimuli with longer ISIs enhances overnight consolidation of a finger-tapping sequence more effectively than applying more stimuli with shorter ISIs. Exploratory electrophysiological analyses revealed that the behavioral response was positively correlated with the number of stimuli with auditory evoked K-complexes relative to the number of stimuli without K-complexes. PTAS stimuli with longer ISIs (>1.25) were associated with a higher likelihood of K-complex responses and increased spindle power. Our findings demonstrate the feasibility of mobile, at-home PTAS combined with app-delivered behavioral tasks in healthy participants and can inform the development of more effective memory enhancement protocols.

## Teaser

**Statement of Significance** Phase-targeted auditory stimulation (PTAS) holds great promise for non-invasively enhancing essential functions of slow-wave sleep. However, current protocols have produced variable results and are often confined to laboratory settings. Our study demonstrates the feasibility of an at-home application of automated PTAS and provides experimental evidence that prolonging interstimulus intervals may positively affect overnight procedural memory consolidation via K-complexes and sleep spindles. Together, our findings lay a foundation for a broader application of PTAS in longitudinal clinical studies to improve patient care and recovery outcomes.

## Introduction

Sleep is essential to our health and well-being. Therefore, it is no surprise that sleep monitoring and modulation techniques are gaining popularity. As mobile recording devices have become more reliable and technically sophisticated [1–5], high-quality sleep data can be obtained in a home setting with high ecological validity, cost-effectiveness, and user comfort. In combination with wearable recording devices that the participants can apply independently, the home setting provides ideal conditions for sleep monitoring over extended periods [2, 6, 7]. Thus, mobile technology holds great promise for longitudinal clinical research [8]. However, ensuring mobile technology’s accessibility and physiological validity in healthy controls is an important next step to advancing its future clinical applicability.

Up-phase targeted auditory stimulation (up-PTAS) has become a valuable tool for modulating sleep non-invasively without disrupting the natural sleep architecture [9, 10]. Up-PTAS relies on applying short (50 ms) bursts of pink noise at the up-phase of ongoing slow oscillations [11]. These stimuli are thought to induce rapid and synchronous neuronal depolarization via bottom-up processes—a response that closely resembles the generation of a K-complex [10]. Functionally, this K-complex can be considered equivalent to a stimulus-evoked slow oscillation (<1 Hz), which is thought to participate in the orchestration of thalamic spindles (oscillatory bursts of 11–16 Hz) and hippocampal ripples to the depolarizing up-phase [11]. This precise temporal coordination of sleep oscillations is assumed to be critical for memory consolidation [12, 13].

Indeed, the enhancement of low (0.5–1 Hz) slow-wave activity (SWA), along with changes in spindle power, has been directly associated with improved overnight declarative memory consolidation in studies that applied up-PTAS [11, 14]. Thus, in principle, PTAS holds great promise for boosting the memory function of sleep, from which clinical populations may profit. However, there seem to be inconsistencies between these studies. A recent meta-analysis has confirmed that up-PTAS positively affects declarative memory overall, but the effect sizes are small and vary considerably across studies [15]. Moreover, the few studies that have examined other domains, such as procedural memory and executive functions, offer conflicting evidence [16–19]. These inconsistencies may be caused by variations in PTAS protocols leading to different evoked electrophysiological and behavioral responses. Such inconsistencies highlight the need for systematic comparisons of PTAS protocols to assess the impact of varying stimulation parameters.

Two studies comparing up-PTAS protocols systematically found no advantage of continuous stimulation over intermittent approaches, such as stimulation in isolated pairs with fixed interstimulus intervals (ISIs) or stimulation in ON–OFF windows, in low SWA enhancement or memory retention, which suggests a self-limiting mechanism [6, 14]. A recent study from our laboratory showed that up-phase targeted stimuli played in short succession tend to induce a long SWA plateau, whereas isolated stimuli reliably evoke a K-complex response, irrespective of the targeted phase [20]. This observation agrees with early reports of evoked K-complexes being susceptible to habituation or refractory processes [21–23]. Interestingly, the K-complex response in our study was accompanied by spindles that were preferentially nested in its up-phase. This induced spindle nesting was positively correlated with the overnight retention of previously learned word pairs [20]. Together, these findings suggest that PTAS for memory consolidation may benefit from an approach that considers K-complex refractory periods, which should allow fewer stimuli to evoke full electrophysiological responses. Therefore, we propose the ISI as an important stimulation parameter in PTAS, the effects of which were evaluated in this study.

We conducted a study in the home setting, using a mobile electroencephalography (EEG) device, the Tosoo Sleep Band Axo (TSB Axo; Tosoo AG, Zurich, Switzerland), to apply two up-PTAS protocols with long or short ISIs and test their effects on the consolidation of a tablet-delivered finger-tapping task (FTT). The FTT is an explicit motor task that has been collectively reported to benefit from sleep-dependent consolidation [24]. It was particularly suited for the home setting due to its high level of standardization and automation. We hypothesized that using this setup, our experimental at-home up-PTAS protocol combined with an app-based FTT would be both feasible and valid and thus would replicate previous in-lab findings. Furthermore, extending previous work [6, 14], we hypothesized that the long ISI up-PTAS protocol would drive behavioral responses more efficiently than the short ISI protocol, by producing at least similar effects with fewer stimuli. To gain a mechanistic understanding, we explored how these systematic variations in the ISI influenced electrophysiological effects and focused specifically on stimulus-evoked K-complexes. Our results confirm the feasibility of PTAS studies in the home setting and provide the first experimental evidence on how ISI length impacts PTAS efficacy and behavior.

## Materials and Methods

### Participants

Eighteen healthy young adults (mean age: 27 ± 5.16 years, eight male) were recruited through an online advertisement on a university platform and word of mouth from March to December 2022. Candidates completed an anonymous questionnaire that assessed the inclusion criteria, including German proficiency at the C1 level or higher, right-handedness, good self-reported sleep quality and regular sleep–wake cycle, good general health, and the ability to comprehend and adhere to study procedures. Candidates were excluded if they reported psychiatric, neurologic, or physical disorders, hearing, or uncorrected vision impairments, skin allergies, shift work, or had traveled through more than one time zone in the past month. To reduce practice effects on the study tasks, candidates were also excluded if they engaged in gaming more than four times a week, were professional musicians, or had previously participated in similar sleep studies. Participants selected for the study agreed to limit caffeine consumption to a maximum of two standard portions per day, abstain from alcohol during assessment days, and from cannabis and other drugs throughout the study. All participants gave written informed consent. The study protocol followed the guidelines provided by the Declaration of Helsinki and was approved by the Cantonal Ethics Committee (BASEC2021-02486).

### Experimental protocol

**Figure 1:**
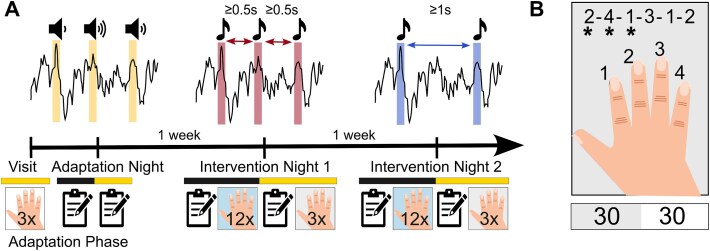
Study and task protocols. (A) A representative participant’s timeline. Icons represent the finger-tapping task with the corresponding number of blocks and questionnaires completed during the study visits and in the evening and morning surrounding the stimulation nights. During the adaptation night, the optimal stimulation volume was determined. During intervention night 1 and intervention night 2, a long ISI (ISI ≥ 1 s) or a short ISI (ISI ≥ 0.5 s) up-phase targeted auditory stimulation (up-PTAS) protocol was administered in consecutive windows of 6 s ON and 6 s OFF. (B) Schematic representation of the tablet-delivered finger-tapping task, where asterisks tracked the current position in the sequence in real time. A representative task block is shown below, with a 30 s active and a 30 s break period. The hand illustration was adapted from *Freepik* Premium resources.

The protocol consisted of an adaptation phase and two intervention nights separated by one week, as shown in Figure 1. All study procedures were performed in the home setting. During the adaptation phase, a study member visited the participants at home to train them in the study tasks and procedures. Following the home visit, participants underwent one to two adaptation nights with a mobile, wearable PTAS device (TSB Axo). The adaptation phase was followed by a wash-out phase of ~1 week. On intervention nights, two stimulation protocols were applied in random order. The two intervention nights had identical timelines. Starting 2 h before bedtime, participants completed a diary, the Karolinska Sleepiness Scale [25] (KSS), the psychomotor vigilance task [26] (PVT), and the FTT, all presented within a tablet-based test battery. As part of a piloting procedure, an unsupervised word-pair task was presented to the first six participants between the FTT and PVT. Data from this task were not included in the analyses. A digital checklist helped participants mount the PTAS device. In the morning, participants removed the device aided by a digital checklist, completed a sleep diary and the KSS once again, and performed retrieval tests for the FTT. To minimize the impact of sleep inertia, participants were instructed to wait at least 30 min after waking before commencing their tasks. All nights were scheduled according to the participants’ habitual sleep and wake times. Participants’ adherence to the protocol was ensured by detailed schedules and information material, regular phone calls, and remote data monitoring.

### Behavioral assessments

Procedural memory consolidation was assessed using a tablet version of the FTT, adapted from Karni et al. [27] Participants were shown a six-digit sequence on a 10.1″ tablet screen (Huawei MediaPad T5) and were instructed to tap it with their dominant hand as fast and accurately as possible. The sequence, consisting of numbers 1–4 corresponding to the index, middle, ring, and pinky fingers, was displayed in the upper half of the screen, with a marker indicating the participant’s current position. The interface included four rectangular buttons on the lower half of the screen, which were customized for each participant to ensure a comfortable hand position. Haptic feedback was provided upon button presses to simulate tactile response. Participants completed the task while seated in a calm environment with the tablet lying flat on a table. They first trained in an easy sequence (1–2–3–3–4–4) for three trials during the adaptation visit to familiarize themselves with the task. Each trial consisted of blocks of 30 s of tapping and a 30 s break to prevent motor fatigue. In the evening session, participants practiced a novel sequence (2–4–1–3–1–2 or 2–1–3–1–4–2) for 12 trials using the same block-wise design. The same sequence was tested in three retrieval trials in the morning session. Sequences were never used twice in the same participant, randomized across stimulation conditions, and matched in difficulty.

We quantified performance and variability measures similarly to previous studies [28–31]. A performance score per trial was calculated as the percentage of correct sequences divided by the intertap interval in seconds, to account for the speed–accuracy tradeoff [32, 33]. Tapping variability was determined as the average standard deviation of intertap intervals in completed sequences per trial [28, 31]. Lower variability indicates more stable timing between taps, which is expected to accompany increased motor skill [34, 35]. A reduction in tapping variability could reflect the adoption of the most effective motor routine [34, 35] or the binding of response chunks into a unified representation [31, 36, 37].

To assess objective and self-reported vigilance, participants completed a standard 10-min PVT and the KSS in the evening before and morning after stimulation nights. In the PVT, participants were presented with a red rectangular box on the tablet screen and were instructed to tap on the screen as soon as a yellow stimulus counter appeared. When a response was recorded, the counter stopped to display the reaction time (RT) in milliseconds. We assessed the response speed (1/RT) in nonlapse trials (RT < 500 ms) as a marker of sustained attention.

### Sleep recordings

EEG, electrooculography (EOG), and electromyography (EMG) were recorded with the TSB Axo, a mobile PTAS device equipped with integrated over-ear headphones for stimulus presentation. Signals from the frontal EEG channel (Fpz), two EOG channels, and two EMG channels were referenced to the right mastoid with the left mastoid as ground. Electrodes were single-use and auto-adhesive (Neuroline 720, Ambu A/S, DK), and their impedance was automatically assessed at the beginning of every recording (average impedance: 90.17 ± 69.45 kOhm). Signals were recorded at a 250 Hz sampling rate, subjected to a real-time anti-aliasing filter and a 50 Hz notch filter, and piped to the built-in stimulation algorithm. The algorithm distinguishes NREM (stages N2 and N3) from non-NREM sleep (wake, N1, and REM sleep) in real time by elevated power in the low delta (2–4 Hz) and high delta (3–5 Hz), and low power in the high beta (20–30 Hz) band in the preceding 80 s of the EEG signal [4]. Power thresholds were derived from historical data of a similar age cohort. Stimulation began after 10 min of stable NREM sleep were detected. A phase-locked-loop algorithm [4] was then enabled to detect slow waves in real time and trigger stimuli (50 ms bursts of pink noise) at the target phase of 45°, corresponding to the down-to-up transition of the ongoing slow wave. Notably, the algorithm prevents stimulation immediately after arousals by detecting short-term (within the past second) increases in beta power, which are indicative of alertness or movement [38]. Stimulation stopped 2.5 h after the first stimulus, leaving the second half of the night without further stimulation. This procedure was introduced to mitigate the effects of PTAS on REM sleep, which has been reported to affect mood [6]. Stimulation was applied in a 6 s ON/6 s OFF design. Stimulation flags in OFF windows (sham stimuli) were reconstructed offline according to the algorithm’s detection criteria.

### Adaptation night stimulation protocol

Previous studies reported a correlation between stimulus response and memory consolidation after PTAS [39]; accordingly, we leveraged the adaptation nights to individualize the stimulation volume to a level that evoked quantifiable PTAS responses without arousing the participant. For this purpose, the individual hearing threshold in the awake state was used to derive a set of four volumes at which stimuli were randomly played during the adaptation night. We selected the lowest volume level at which a visible auditory evoked potential (AEP) could be evoked. If the optimal stimulation volume could not be determined after one adaptation night, a second night was recorded. The volume defined in the adaptation phase was used consistently throughout the intervention nights. The final volumes ranged from 46 to 64 dB sound pressure level.

### Intervention night stimulation protocols

During intervention nights, two up-PTAS protocols were administered in random order: a short ISI protocol and a long ISI protocol. Both protocols shared the same stimulation period, an ON–OFF block design, and a consistent stimulation volume derived from the adaptation phase but differed systematically in the ISIs. In the short ISI protocol, we selected an ISI of ≥0.5 s with the expectation of stimulating every slow wave detected within ON windows once. In the long ISI protocol, we extended the ISI to ≥1 s with the expectation of skipping certain slow waves within a train or only stimulating consecutive waves in the slow oscillation frequency range (≤1 Hz).

### Sleep scoring and artifact rejection

For sleep scoring, signals were filtered, re-referenced, and scored according to AASM guidelines [40]. To filter the signal, third-order Butterworth digital IIR filters were applied forward and backward to avoid phase shifts. The signal from Fpz was band-pass filtered between 0.5 and 35 Hz. The signals from the two eye channels were band-pass filtered between 0.3 and 35 Hz. Signals from the two muscle channels were band-pass filtered between 10 and 100 Hz and re-referenced bilaterally if visually assessed signal quality was good in both channels. After filtering, all signals were downsampled to 128 Hz. Vigilance states (wake, N1, N2, N3, REM) were visually scored by two sleep experts in 30 s epochs in *Visbrain* [41] and validated by a third sleep expert. All scorers were blind to the experimental condition and the stimulation flags. After scoring, NREM (N1, N2, N3) sleep epochs were subjected to a semi-automatic artifact detection routine adapted from Leach et al. [42] for single-channel signals.

### E‌EG signal preprocessing

The EEG signal was first subjected to a low-pass filter with a pass band of up to 30 Hz (−6 dB attenuation at 39.86 Hz; filter order: 46, at a sampling rate of 250 Hz) then subjected to a high-pass filter with a pass band starting at 0.5 Hz (−6 dB attenuation at 0.37 Hz; filter order: 2988, at a sampling rate of 250 Hz), similar to a previous study at our laboratory [20]. Filters were Kaiser window FIR filters with zero-phase shift to avoid phase distortions. Filtering and all consecutively described signal processing steps were performed in MATLAB R2022a (The MathWorks Inc., 2022) using EEGLAB [43] and the FieldTrip toolbox [44].

### Quantification of K-complexes

Stimulus-associated K-complexes were detected in the preprocessed Fpz signal with the *DETOKS* algorithm [45]. Briefly, the algorithm first removes transients from the signal and then splits it into a low-frequency component and an oscillatory component. A Teager-Kaiser energy operator (TKEO) is applied to the low-frequency component for detecting K-complexes and to the oscillatory component for detecting spindles. TKEO thresholds must be manually adjusted and were optimized on this data set to obtain the maximal number of events in NREM epochs but a minimal number of events in artifactual epochs.

Before subjecting the Fpz signal to the *DETOKS* algorithm for K-complex detection, we automatically removed pulse artifacts from the data with a published algorithm for single-channel data (*brMEGA*) [46]. The low-pass filter cutoff for the low-frequency component was left at the default value (4 Hz), but we extended the duration threshold for K-complexes to 0.5–2 s, corresponding to a frequency range of 0.5–2 Hz, for comparability with previous reports [47, 48]. Stimulus-associated K-complexes were defined to start within 1.5 s after stimulus onset and were determined separately for ON stimuli and sham stimuli. The final detection result was visually validated against the preprocessed EEG signal. Furthermore, we compared the average waveform and time-frequency response across detected events (2 s epochs, aligned at K-complex start) with a random selection of NREM sleep trials without K-complexes. Time-frequency responses were assessed according to the procedure described in Time-frequency analysis.

### Determination of ISI bins

To determine in greater detail at which ISI stimulus-associated K-complexes were most likely to occur, we defined ISI bins using a data-driven approach. Defining such a uniform scale was necessary because ISIs were not fixed across the sample but varied highly depending on the underlying slow waves. ISIs preceding auditory stimuli were determined across the sample and split into roughly uniformly distributed bins (Supplementary Figure S1). This procedure produced a final set of ten ISI bins: 0.5–0.75 s, 0.75–1 s, 1–1.25 s, 1.25–1.5 s, 1.5–2 s, 2–2.5 s, 2.5–6.5 s, 6.5–8 s, 8–10.5 s, and >10.5 s. We then collapsed all available nights per individual and quantified the number of stimuli followed by a K-complex and the number of stimuli followed by no K-complex per ISI bin for each participant and defined event versus no event as a binary outcome.

### Time-frequency analysis

We set the ISI bin where K-complex probability plateaued as the threshold to categorize stimuli by their preceding ISI into above-threshold and below-threshold stimuli. Four-second epochs surrounding ON stimuli were contrasted with epochs surrounding sham (OFF) stimuli of the same stimulus category. For contrasts between stimulus categories, we contrasted the ON epochs. The number of epochs contrasted per night was matched by randomly sampling epochs from the majority class.

The AEP of each stimulus category was determined by averaging across all ON and OFF epochs separately and then calculating the difference between ON and OFF. The time-frequency response of each stimulus category was obtained by convolving the signal in ON and OFF epochs with a range of Morlet wavelets covering a linearly increasing frequency space from 1 to 30 Hz in 1 Hz steps, with a linearly increasing cycle number from 3 to 17.5. Average time-frequency power maps were log-normalized by a whole-epoch baseline calculated separately for each frequency bin across all ON and OFF epochs.

To assess statistical differences between ON and OFF epochs, we used a standard nonparametric clustering procedure [49]. Following the computation of the real *t* map (paired Student’s *t*-tests, two-tailed, α = 0.05), reference *t* maps were obtained by randomly permuting (*n* = 2000) ON and OFF epochs within each night and performing a paired *t*-test on the permuted time-frequency maps. Cluster thresholds were defined based on the 95th percentile of clusters of significant *t* values in reference maps and used for cluster correction. An equivalent cluster correction was applied to the *t* time series resulting from ON–OFF AEP contrasts (paired two-tailed Student’s *t*-tests, α = 0.05). Cluster permutation statistics were performed in MATLAB. The same approach was used to contrast ON epochs across the two stimulus categories.

### Phase-amplitude modulation analysis

To assess the coupling strength between the fast spindle (13–16 Hz) band and the underlying wave in the slow oscillation frequency range (1 Hz) within 2 s after the stimulus, we computed the *z*-scored modulation index (zMI) [50] separately per night and stimulus category. The empirical MI was *z*-transformed with the null distribution, which was constructed by repeatedly (*n* = 200) cutting the sigma-epochs at a random sample and reversing the order of both parts [51]. For this analysis, we used 18 phase bins of 20°, similar to previous studies [50]. The frequency range for the sigma band was determined from previous reports of fast spindles being preferentially coupled to the up-phase of slow oscillations and, therefore, potentially relevant to sleep-dependent memory processes [52]. The frequency of the underlying wave was determined to match the frequency ranges reported for K-complexes [53, 54] and previous reports that found most spindle coupling in the up-phase of the 1 Hz oscillation [20, 55].

### Statistics

Statistical analyses included paired parametric and nonparametric tests for comparing variables between the stimulation conditions and stimulus categories and robust linear mixed-effects models with random intercepts for participants (*robustlmm* package in R [56]) for assessing the linear relationship between variables of interest and behavioral outcomes. Normality of residuals for paired tests was evaluated by histogram, Q–Q plot, and Shapiro–Wilk test. If the criteria for normal distribution were met, the comparison was performed with a two-sided Student’s *t*-test. Otherwise, we chose a nonparametric two-sided Wilcoxon signed-rank test. Outliers were identified at a threshold of |*z*| > 3. For correlations concerning only one condition, we used robust percentage bend correlations [57], an approach that down-weights a specified percentage (20 per cent) of marginal observations deviating from the median, before computing Pearson’s correlation on the transformed data. Statistical significance was set at *p* < .05. Effect sizes for pairwise tests were reported as Hedge’s *g*. The *p*-values for robust linear mixed-effects models were approximated based on the *t* statistic and residual degrees of freedom reported in the model output. Unless stated differently, all statistical analyses were performed in R Statistical Software (v4.4.1; R Core Team, 2024).

## Results

### Feasibility of automated auditory stimulation in the home setting

We conducted a randomized double-blind cross-over study subjecting 18 healthy participants to two different up-PTAS protocols and a series of behavioral tasks in the home setting. Adherence to the protocol was monitored in several ways. Firstly, data quality was assessed daily after the data were transmitted automatically from participants’ devices to the server. Secondly, time stamps of task completion were compared to the participants’ individual schedules. Overall, 16 participants had complete EEG data from both intervention nights; these data were used for EEG analyses that did not involve associations with FTT data. Of this subsample, 14 participants had complete FTT data. Two participants’ EEG data from the long ISI stimulation night was lost for technical reasons, and two participants were excluded from behavioral analyses for protocol deviations.

We first assessed the impact of the protocols on sleep architecture, vigilance (PVT), and subjective sleepiness (KSS). Of the 16 participants with complete EEG data, KSS data were available in 15 individuals and complete PVT data in 13 individuals. No significant differences were observed between stimulation conditions (Table 1) except for a higher percentage of N1 sleep in the long ISI condition (*g* = 0.48*, p* = .035), which was driven by differences in the unstimulated part of the night (Supplementary Table S1). This suggests that the effect on N1 sleep may not be directly related to the stimulation. Arousal rates did not differ significantly between conditions (see Supplemental Analyses and Supplementary Table S2). In summary, we found no differential effects of the two protocols on self-reported variables, and objective sleep markers were largely similar.

**Table 1:** Comparison of sleep architecture, KSS, and PVT outcomes between the stimulation conditions

Variable (units)	Short ISI (mean ± SD)	Long ISI (mean ± SD)	Mean difference	95% CI	*g*	*P*
TTB (min)	468 ± 46.6	485 ± 6.7	17.3	[−15.0; 49.5]	0.39	.271
TST (min)	399 ± 54.8	396 ± 46.4	−3.4	[−24.5; 17.7]	−0.06	.733
SL (min)	31 ± 23.2	39 ± 46.0	7.9	[−7.4; 23.3]	0.14	.288
SEF (%)	89.8 ± 6.38	87.9 ± 9.88	−1.90	[−4.62; 0.83]	−0.17	.159
WASO (min)	14 ± 9.6	24 ± 30.4	9.6	[−5.4; 24.6]	0.36	.194
REML (min)	79 ± 26.9	104 ± 57.0	25.0	[−1.8; 51.8]	0.49	.065
N1 (%)	4.7 ± 2.05	6.0 ± 2.87	1.32	[0.11; 2.53]	** 0.48**	**.035**
N2 (%)	45.0 ± 7.82	42.0 ± 8.16	−2.98	[−6.79; 0.83]	−0.35	.116
N3 (%)	19.7 ± 5.87	18.9 ± 4.79	−0.77	[−2.94; 1.40]	−0.13	.462
REM (%)	20.5 ± 4.79	21.0 ± 6.34	0.54	[−2.11; 3.19]	0.09	.669
Wake (%)	10.2 ± 6.39	12.1 ± 9.88	1.89	[−0.83; 4.62]	0.17	.159
ΔKSS	−0.5 ± 1.77	−0.2 ± 2.18	0.27	[−0.79; 1.32]	0.13	.597
Morning KSS	3.4 ± 1.30	3.8 ± 2.04	0.40	[−0.53, 1.33]	0.21	.373
ΔPVT	0.13 ± 0.20	0.06 ± 0.11	−0.07	[−0.16; 0.02]	0.38	.129
Morning PVT	2.84 ± 0.36	2.79 ± 0.35	−0.06	[−0.13; 0.01]	0.16	.093

Mean differences refer to the contrast long ISI > short ISI. The reported *p* values were obtained by paired Student’s *t*-tests (*N* = 16) and effect size is reported in terms of Hedge’s *g*. Significant comparisons are marked in bold. Abbreviations: TTB, total time in bed; TST, total sleep time; SL, sleep latency; SEF, sleep efficiency; WASO, wake after sleep onset; REML, rapid eye movement sleep latency; N1, NREM sleep stage 1; N2, NREM sleep stage 2; N3, NREM sleep stage 3; REM, rapid eye movement sleep; ΔKSS, overnight change in Karolinska Sleepiness Scale; ΔPVT, overnight change in psychomotor vigilance task response speed (1/*s*).

To evaluate whether the ISI manipulation was successful, we quantified the stimulation characteristics in each condition. The overall number of stimuli applied per night ranged from 186 to 2028, with an average of 1098 ± 501 in the short ISI condition and 638 ± 260 in the long ISI condition. The total number of stimuli was significantly lower in the long ISI condition than the short ISI condition (*g* = −0.98*, p* < .001). Accordingly, the long ISI condition showed a significantly longer median ISI within ON windows (long ISI: Mean = 1.92 ± 0.13 s, short ISI: Mean = 1.15 ± 0.14 s, long ISI > short ISI: *g* = 5.36, *p* < 1 × 10^−9^). Together, these data show that the two protocols were clearly distinguishable by their ISIs.

### Effects of auditory stimulation differences on finger-tapping performance

Participants were assessed on the FTT before and after each stimulation night. They completed 12 learning trials of a novel sequence in the evening and three retrieval trials of the same sequence the following morning. We quantified procedural learning with a performance score and tapping variability, as in previous studies [28–31]. Equivalent analyses of other performance metrics, including accuracy and speed as individual contributors to the performance score and the number of correct [58, 59] and finished sequences [59, 60], are shown in Supplementary Figure S2. The patterns observed in these metrics were consistent with the effects subsequently reported for performance scores and tapping variability.

**Figure 2:**
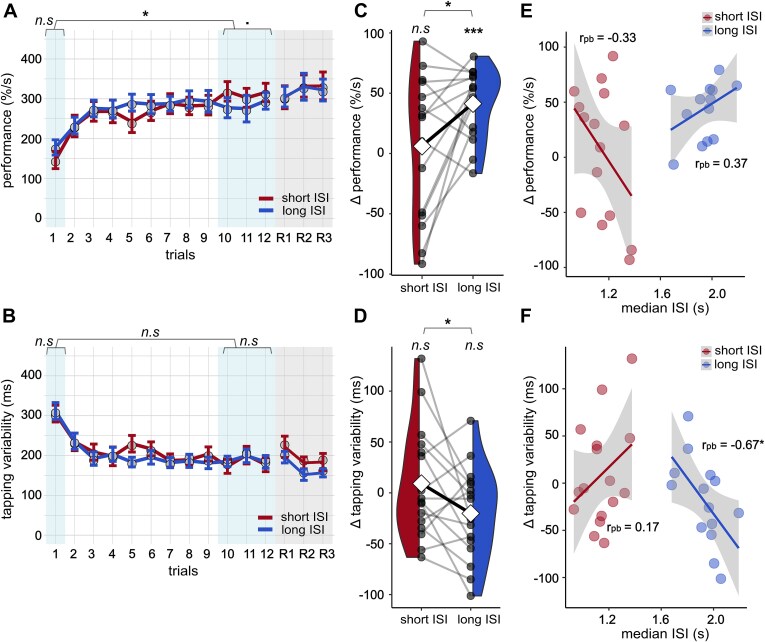
Learning and consolidation of the finger-tapping task between long ISI and short ISI conditions. (A) and (B) Trajectories of performance and tapping variability across learning (1–12) and retrieval (R1–R3) trials. Circles indicate means across participants and error bars indicate standard errors of the mean. Shaded areas represent the baseline (trial 1), learning plateau (trials 10–12), and retrieval (trials R1 - R3). Asterisks represent significant differences in baseline, learning rate, and plateau with ^*^p < .05, n.s. not significant; paired two-tailed Student’s *t*-test. (C) The overnight change in performance was significantly different between short ISI and long ISI conditions (*p* < .05, *N* = 13, paired two-tailed Student’s *t*-test). One outlier was removed from the comparison for being >3 SDs away from the population mean. (D) the overnight change in tapping variability was significantly different between short ISI and long ISI conditions (*p* < .05, *N* = 14, paired two-tailed Student’s *t*-test). In (C) and (D), diamonds indicate condition means, bold line indicates mean difference, circles represent individual participants, and individual lines represent within-subject differences. Asterisks above violin plots represent significant differences in the performance metric at retrieval versus at plateau (^***^p < .001, n.s. not significant), calculated separately for each condition, while horizontal brackets refer to differences in overnight gains between conditions. (E) Overnight change in performance in the FTT by condition and median ISI with linear model fit. Only the main effect of median ISI (β = −216.27, *t* = −3.13, *p* = .005) was significant, but not the interaction of condition and median ISI (β = 65.18, *t* = 0.61, *p* = .548). Robust percentage bend correlations were not significant in any condition. (F) Overnight change in tapping variability in the FTT by condition and median ISI with linear model fit. The interaction of condition and median ISI was significant (β = −285.04, *t* = −2.013, *p* = .047). Robust percentage bend correlations indicated a significant association between ISI and overnight change in tapping variability only in the long ISI condition. In (E) and (F), solid lines indicate the model prediction, shaded areas the 95% Cl.

Figure 2A shows that participants exhibited a logarithmic learning trajectory during the training session. Their performance scores increased rapidly during the first three trials and then approached a plateau. Likewise, participants exhibited an inverse logarithmic learning curve for tapping variability, with rapid reductions during the first three trials and a plateau in later trials (Figure 2B). The learning plateau for each participant and condition was approximated by the average of the last three learning trials in the evening session. The learning rate was approximated by the difference between the first trial, considered the baseline, and the learning plateau. For tapping variability, these markers of pre-stimulation learning did not differ significantly between conditions, even after controlling for order effects expressed as Night and Night$\times$ Condition interaction. However, we found significant differences in performance score learning rate (*g* = −0.74, *p* = .024) and a trend-level difference in plateau performance (*g* = −0.41, *p* = .054), which became significant when controlling for Night and Night $\times$ Condition interaction in robust linear mixed-effects models (baseline performance: β = 31.40*, t* = 3.36, *p*=.003, plateau performance: β = −34.66, *t* = −2.13, *p* = .044; learning rate performance: β = −61.61, *t* = −3.87, *p* < .001). These results indicate that at the stage of learning, tapping variability evolved similarly across conditions, whereas performance scores exhibited differences between conditions even before the PTAS intervention.

Next, we assessed whether overnight performance changes differed between the stimulation conditions. Overnight performance change was defined as the difference between the learning plateau and the average of the three retrieval trials, as established in prior work [59]. Performance scores increased significantly more in the long ISI condition than in the short ISI condition (*g* = 0.81, *p* = .022*;* long ISI: mean overnight change = 44.12*, p* < .001*,* short ISI: mean overnight change = 0.90, *p* = .959), after excluding one outlier from the sample (see Statistics section for outlier definition; Figure 2C). This outlier was excluded from all subsequent analyses related to the performance score.

Given that a pre-stimulation difference in performance scores was observed between conditions, we fitted a separate robust linear mixed-effects model to control for plateau performance and its interaction with condition (Supplemental Analyses). The dependent variable in this case was the performance score at retrieval. As expected, the main effect of plateau performance was significant (β = 0.98, *t* = 4.721,  *p* < .001), indicating that retrieval performance increased with the performance level achieved during prior learning. The model indicated no significant contribution of the Plateau $\times$ Condition interaction to retrieval scores, whereas the condition effect remained significant (β = 39.95, *t* = 2.225,  *p* = .037; Supplementary Table S3).

Likewise, overnight tapping variability significantly differed between the long and short ISI conditions (*g* = −0.64*, p* = .039), with a decrease observed in the long ISI condition (mean overnight change = −19.09, *p* = .146) and a slight increase in the short ISI condition (mean overnight change = 15.81, *p* = .310; Figure 2D). Control analyses confirmed that these effects were unlikely to be driven by order effects or the distribution of sleep stages (Supplemental Analyses and Supplementary Tables S4 and S5). Notably, the long ISI stimulation benefited overnight performance changes despite the lower number of stimuli than the short ISI stimulation.

We fitted a robust linear mixed-effects model for each behavioral outcome to assess whether the ISI manipulation within ON windows predicted the behavioral differences observed between the stimulation protocols. The model included median ISI, condition, and Median ISI $\times$ Condition as fixed factors and participant as a random intercept. We found a significant main effect of median ISI (β = −216.27, *t* = −3.13, *p* = .005) on overnight change of performance scores, whereas condition and the interaction effect were not significant (Figure 2E). For tapping variability, the interaction of condition and median ISI was significant (β = −285.04, *t* = −2.01, *p* = .047; Figure 2F), suggesting that ISI effects on tapping variability differ by condition. To assess this in more detail, we determined the percentage bend correlation within each condition. This showed a significant association between median ISI and overnight change in tapping variability only in the long ISI condition (short ISI: *r*_pb_ = 0.17, 95% CI *=* (−0.35, 0.61), *p* = .526; long ISI: *r*_pb_ = −0.67, 95% CI = (−0.89, −0.22), *p* = .017; Figure 2F). Correlations between median ISI and overnight change in performance scores were not significant (Figure 2E). These findings suggest that longer ISIs predict the beneficial effects of the long ISI condition on overnight decrease in FTT tapping variability. However, ISI is not a significant predictor of behavioral response in the short ISI condition.

### Electrophysiological stimulus response

To explore the mechanism through which longer ISIs mediate improvements in overnight procedural memory consolidation, we quantified stimulus-evoked K-complexes and their relationship to behavioral outcomes. The difference in low SWA between ON and OFF windows (ΔlowSWA; see Supplemental Analyses) is reported in Supplementary Figure S3 as an alternative, coarser marker of response [20, 61].

The K-complex detection algorithm [45] identified, on average, 9.8 (±2.11) NREM sleep K-complexes per minute in the short ISI condition and 10.0 (±1.86) NREM sleep K-complexes per minute in the long ISI condition, with no significant differences between the conditions. However, in the long ISI condition, K-complex density was significantly increased in N3 sleep within the stimulated part of the night (Supplementary Table S6). This finding may relate to a stimulation effect, because most stimulations were applied during N3 sleep (Supplemental Analyses). On average, 299 (±131.5) and 204 (±90.8) K-complexes were associated with a stimulus in the short ISI and long ISI conditions, respectively. These events exhibited the expected waveform (P200, N550, P900) and time-frequency signature, including increased delta power and reduced high-frequency activity (Supplementary Figure S4), thus confirming their alignment with canonical K-complex characteristics.

**Figure 3:**
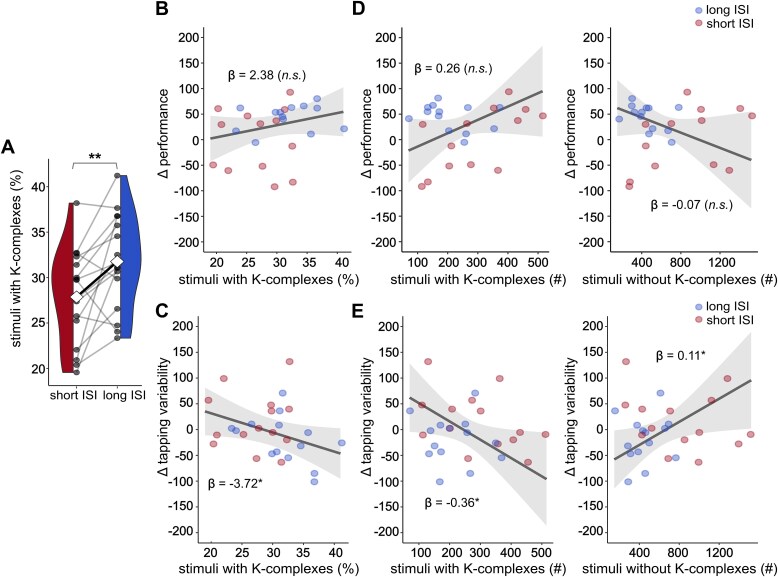
Proportion of auditory stimuli followed by detected K-complexes: comparison between short ISI and long ISI protocols and association with overnight change in tapping variability. (A) The proportion of stimuli followed by a K-complex was significantly higher in the long ISI than in the short ISI condition. Stimulus proportion per night was assessed by normalizing the total number of stimuli followed by a K-complex by the total number of ON-window stimuli administered during that night. White diamonds indicate condition means, bold black line indicates mean difference, gray circles represent individual participants, and gray lines represent within-subject differences. Asterisks represent significance levels with ^**^p < .01; paired two-tailed Student’s *t*-test. (B) Fixed effects plot of robust linear mixed-effects model (rLMM) predicting overnight change in performance from the stimulus proportion followed by a K-complex. The effect of stimulus proportion with K-complexes was not significant (β = 2.38, *t* = 1.31, *p* = .204). (C) Fixed effects plot of rLMM predicting overnight change in tapping variability from the stimulus proportion followed by a K-complex. The effect of stimulus proportion followed by a K-complex was significant (β = −3.72, *t* = −2.11, *p* = .045)*.* (d) Fixed effects plot of rLMM disentangling contributions of stimuli followed by a K-complex and stimuli not followed by a K-complex to overnight change in performance. The individual contributions did not reach significance (stimuli with K-complexes: β = 0.26, *t* = 1.66, *p* = .111; stimuli without K-complexes: β = −0.07, *t* = −1.47, *p* = .155). (E) Fixed effects plot of rLMM disentangling contributions of stimuli followed by a K-complex and stimuli not followed by a K-complex to overnight change in tapping variability. The effects of stimuli with K-complexes and stimuli without K-complexes were significant (stimuli with K-complexes: β = −0.36, *t* = −2.22, *p* = .036; stimuli without K-complexes: β = 0.11, *t* = 2.27, *p* = .033). In (B)–(E), solid lines indicate the model predictions, gray-shaded areas the 95% CI. Asterisks represent significance levels with ^*^p < .05, n.s.: not significant.

As Figure 3A shows, the relative number of stimuli followed by a K-complex was significantly larger in the long ISI condition than in the short ISI condition (*g* = 0.70, *p* = .010). Although the relationship of the proportion of stimuli followed by K-complexes with overnight changes in performance scores failed to reach statistical significance (β = 2.38, *t* = 1.31, *p* = .204, Figure 3B), we found it to be significantly associated with overnight reductions in tapping variability (β = −3.72, *t* = −2.11, *p* = .045; Figure 3C). From a stimulus-centered perspective, the proportion of stimuli followed by K-complexes reflects a tradeoff between the number of stimuli associated with a K-complex and the number of stimuli not associated with a K-complex. To further assess the individual contributions of these variables, we fitted robust linear mixed-effects models with main effects for the absolute number of stimuli with K-complexes and remaining stimuli without K-complexes. Figure 3D shows this model for the performance score, where we found no significant effects of either variable. However, for the overnight change in tapping variability, we found opposing contributions of stimuli with K-complexes and stimuli without K-complexes: The number of stimuli with K-complexes was associated with a reduction of tapping variability (β = −0.36, *t* = −2.22, *p* = .036), whereas the number of stimuli not followed by a K-complex was associated with an increase of tapping variability (β = 0.11, *t* = 2.27, *p* = .033, Figure 3E).

Overall, the results suggest that long ISI stimuli evoke K-complexes more efficiently than short ISI stimuli. The proportion of stimuli with relative to stimuli without a K-complex response may contribute to the behavioral effects in the FTT. Of note, the short ISI condition had both significantly more stimuli with K-complex responses and stimuli with no K-complex responses than did the long ISI condition (Supplementary Table S6). This result confirms that the absolute number of evoked K-complex responses is less crucial to behavior than their proportion to stimuli with no K-complex response.

### Relationship of ISI and electrophysiological stimulus response

**Figure 4:**
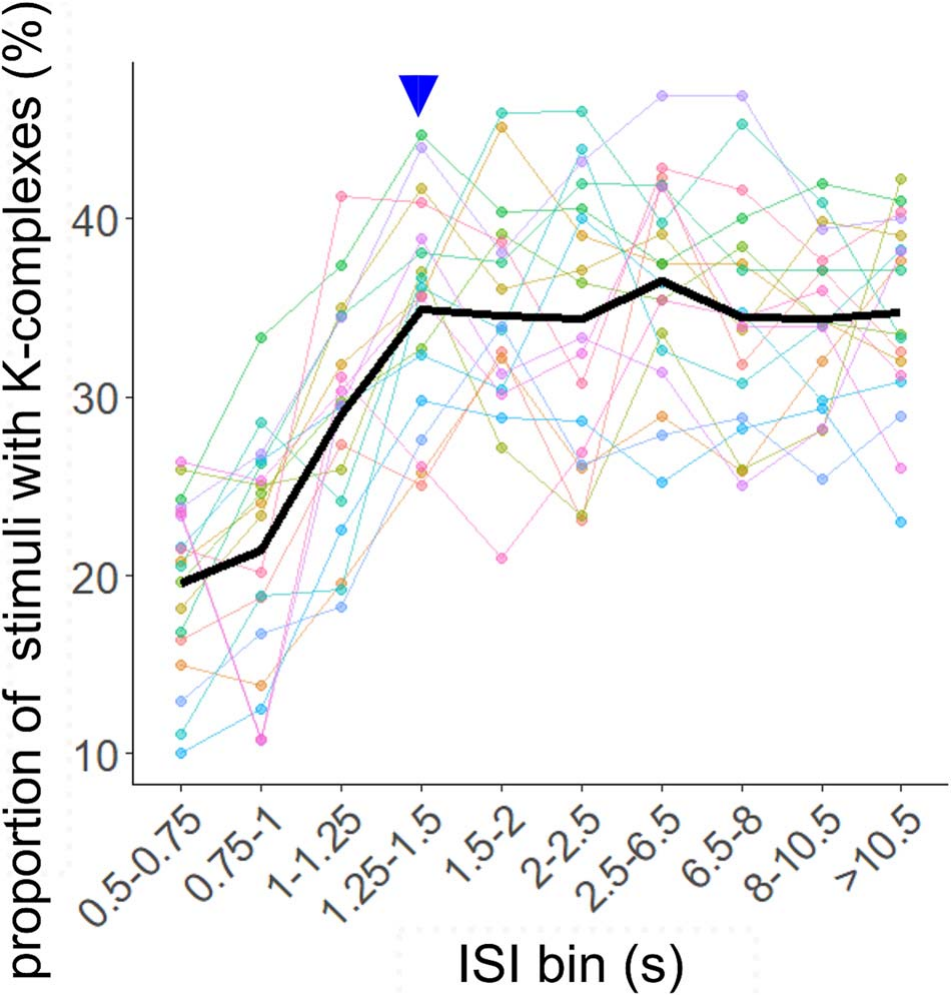
Proportion of stimuli followed by a K-complex by ISI bin. The normalization was performed with all stimuli per ISI bin, pooling short ISI, and long ISI nights per participant. The bold line represents the average across participants; finer lines represent individual trajectories. The arrow marks the bin at which the proportion of stimuli with a K-complex stabilized.

We assessed the relationship between ISI and the likelihood of stimulus-associated K-complexes to determine the optimal range of ISIs for evoking K-complex-like responses. As Fig. 4 shows, the relative number of stimuli followed by a K-complex increased linearly across the lowest ISI bins and stabilized at ~35 per cent starting at the bin spanning ISIs of 1.25–1.5 s. Individual lines showing the collapsed data from both intervention nights per participant indicate that all individuals followed a similar trajectory, with some variance in start and end levels. Overall, these results suggest that stimuli preceded by longer ISIs are more likely to evoke K-complex-like responses than stimuli preceded by shorter ISIs. Moreover, the fact that the proportion of stimuli followed by K-complexes plateaus at ISIs > 1.25 s suggests that such a response should be evoked most reliably at an ISI threshold of >1.25 s.

**Figure 5:**
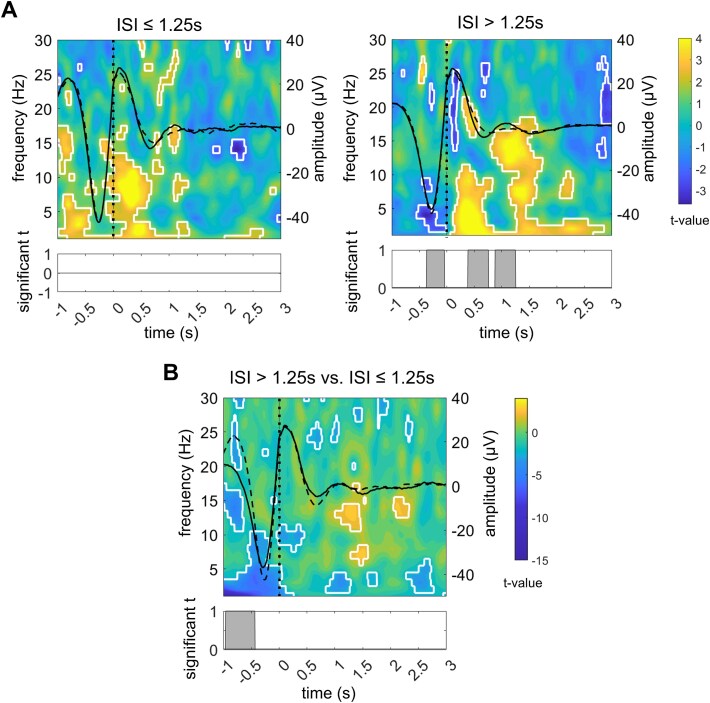
Responses to auditory stimuli categorized according to ISI cutoff. (A) *t* maps relating to power in time-frequency space contrasting ON- and OFF-window stimuli classified by ISI. White margins indicate significant clusters after permutation-based cluster correction (paired two-tailed Student’s *t*-tests, α = 0.05). Solid black lines represent average waveforms of stimulus-locked responses in ON windows. Dashed black lines represent average waveforms of stimulus-locked responses in OFF windows. Significant differences between the two waveforms are marked in the bottom plot (paired two-tailed Student’s *t*-tests with permutation-based cluster correction, α = 0.05). Vertical dashed lines represent stimulus onset. (B) *t* map relating to power in time-frequency space contrasting ON-window stimuli of the two ISI categories (above-threshold > below-threshold). White margins indicate significant clusters after permutation-based cluster correction (paired two-tailed Student’s *t*-tests, α = 0.05). Solid black lines represent average waveforms of stimulus-locked responses to above-threshold stimuli. Dashed black lines represent average waveforms of stimulus-locked responses to below-threshold stimuli. Significant differences between the two waveforms are marked in the bottom plot (paired two-tailed Student’s *t*-tests with permutation-based cluster correction, α = 0.05). Vertical dashed lines represent stimulus onset.

To test this prediction, we contrasted time-locked responses to stimuli of two categories: those preceded by an ISI > 1.25 s (above-threshold stimuli) and those preceded by an ISI ≤ 1.25 s (below-threshold stimuli). Data were collapsed across intervention nights. Figure 5A shows the contrast of stimuli in ON windows with comparable sham stimuli, thus meeting the same ISI criteria, in OFF windows. To directly compare the two stimulus categories, we contrasted above-threshold and below-threshold ON stimuli while balancing epoch counts (Figure 5B). AEPs were not significantly different from each other (Figure 5B), even though in the ON–OFF contrasts, it appeared that only the above-threshold stimuli could evoke significant N550 and P900 components (Figure 5A, right panel). The pre-stimulus waveform had a significantly higher amplitude before below-threshold stimuli than before above-threshold stimuli, which likely reflects an AEP from a preceding stimulus. Below-threshold stimuli were associated with stronger pre-stimulus delta-theta power, immediate post-stimulus alpha enhancement, brief clusters of increased high-frequency activity between 500 ms and 2.5 s, but weaker spindle responses. In contrast, above-threshold stimuli elicited stronger spindle activity centered at ~1.2 and 2.2 s. Judging from the ON–OFF contrasts, the first spindle cluster likely reflects a relatively stronger spindle enhancement after above-threshold stimuli (Figure 5A, right panel), whereas the second spindle cluster can be attributed to a delayed suppression of spindle power after below-threshold stimuli (Figure 5A, left panel).

Notably, the delayed sigma response was time-locked to the up-phase of the acoustically evoked K-complex, which implies the possibility that the phase-amplitude modulation between K-complex and sigma power was also part of the stimulus response. Therefore, we quantified the zMI between the 1 Hz slow oscillation, which falls into the frequency range of the K-complex, and fast sigma power (13–16 Hz) within 2 s after stimulus onset in ON windows. The comparison of stimulus-associated modulation between the slow oscillation and fast sigma power showed no significant differences in coupling strength between above-threshold and below-threshold stimuli (*g* = 0.14, *p* = .405).

## Discussion

We compared two automated up-PTAS protocols with systematic variations of the ISI for their effects on overnight changes in finger-tapping performance in the home setting. The up-PTAS protocol with prolonged ISIs significantly increased performance scores and reduced the tapping variability of a learned finger-tapping sequence after a single night of stimulation more than a protocol with shorter ISIs. The fact that neither metric returned to baseline after a night of stimulated sleep but instead stabilized at levels reflecting improvement suggests the presence of offline gains, indicating a consolidation process [30, 60]. These behavioral benefits were associated with the proportion of stimuli evoking a K-complex response, which was favored by longer ISIs. Specifically, we found that at ISIs > 1.25 s, a plateau was reached at which about 35 per cent of stimuli reliably evoked a K-complex. This response was also marked by an increase in fast sigma power, which was nested in the up-phase of the evoked K-complex. Although we performed this study in a less controlled setting, complete EEG data were recorded in 16 out of 18 participants (89 per cent), and complete EEG and FTT data in 14 out of 18 participants (78 per cent). These results suggest that

(1) automated application of up-PTAS in naturalistic sleep in the home setting both is feasible and influences next-day behavioral responses,
(2) up-PTAS may be optimized for overnight procedural memory consolidation by introducing longer ISIs of at least 1.25 s,
(3) long ISI up-PTAS favors K-complexes and spindles.

In support of our first hypothesis, the participants tolerated the mobile stimulation device well, with no dropouts and good adherence to the sleep protocols. All but two participants completed tasks and questionnaires according to the protocol. In the short ISI condition, we achieved similar stimulation numbers as in a previous study using the same protocol in a laboratory setting [61] and the electrophysiological responses that we recorded replicated previous reports of PTAS-induced stimulation effects [5, 20, 62–64]. Similarly, our tablet-based FFT captured the logarithmic learning curve typically observed in lab-based FTTs [28, 29, 37, 60, 65, 66]. Our sample displayed similar performance ranges as reported by Fattinger et al. [28], suggesting that the tablet FTT is learned in a comparable manner as the keyboard FTT. However, tapping variability was systematically higher in comparison, which may point to different kinematics in tablet and keyboard tapping. Although this factor is unlikely to have affected our within-participant comparisons, comparisons between lab- and home-setting studies should be approached with caution. Previous research has validated the sleep stage and phase specificity of the TSB Axo [67] and replicated established electrophysiological stimulation effects with automated home-based PTAS in various study populations [2, 5–7]. We extended these previous efforts by including an experimental comparison between PTAS protocols to investigate the ISI as a parameter of interest and a behavioral assessment of overnight consolidation of a finger-tapping sequence. This task showed differential effects that were sensitive to the stimulation protocol.

Our second hypothesis predicted that the long ISI up-PTAS protocol would drive overnight performance change more efficiently than the short ISI protocol. In agreement with a study rejecting the idea of a linear relationship between stimulus number and PTAS effects on sleep-dependent memory consolidation [14], the long ISI protocol proved more effective in increasing performance scores and decreasing tapping variability overnight despite involving significantly fewer stimuli. These overnight performance improvements, so-called “offline gains,” were previously observed for the performance score as a consequence of sleep [30]. The overnight decrease in tapping variability observed in the long ISI condition relative to the short ISI condition could indicate more fluent and stable motor performance. This finding aligns with previous observations, according to which sleep selectively improved the speed of the key-press transitions that were the slowest before sleep [37].

Our exploratory analysis suggests that the behavioral effect was mediated by the relative number of stimuli associated with a K-complex. The ISI determined the rate at which K-complexes occurred after stimuli, replicating earlier findings that showed more K-complexes after rare stimuli than after frequent ones [22, 23, 68]. The plateau proportion of stimuli evoking a K-complex at longer ISIs (>1.25 s) was in a similar range (~40 per cent) as reported previously, where single-frequency stimuli at slightly higher intensities and lower rates were employed [22, 69]. Stimuli preceded by shorter ISIs (≤1.25 s) were associated with a lower probability of K-complexes. Stimuli that failed to evoke a K-complex were negatively associated with overnight performance changes, whereas those that did were positively associated with such changes. We demonstrated that stimuli preceded by longer ISIs (>1.25 s) produced a more pronounced spindle response than those preceded by shorter ISIs. Like K-complexes, spindles are known to have a refractory period, which has been estimated at 3–6 s [70]. Interestingly, an earlier study found that stimuli that were presented at a longer latency of ~2.5 s after a spindle, close to the end of the refractory period, enhanced early spindle responses and memory retention significantly more than did stimuli presented shortly after a spindle [70]. In our study, stimuli preceded by shorter ISIs might have fallen into the spindle refractory period and therefore failed to evoke a sigma response within the measurement time window.

The fact that we could not replicate previous observations linking increased coupling strength between fast spindles and slow oscillations with procedural memory consolidation [71] may be related to the low-density electrode montage, which did not capture central-parietal areas, including the motor cortex. In line with this interpretation, a previous study found coupling strength assessed by the modulation index to be augmented at central-parietal locations but to remain relatively low at frontal locations after PTAS [72]. Together, our findings contribute to a body of research suggesting that incorporating longer ISIs in PTAS designs elevates the likelihood of stimulating optimal windows for memory consolidation by K-complex and spindle responses. Although it is currently unclear why stimuli that fail to evoke K-complex and spindle responses should have a negative impact rather than a neutral one, the increase in high-frequency activity triggered by these stimuli points at a potential role of microarousals in this process [73]. Future studies using high-density EEG setups should explore the mechanisms by which spindles, K-complexes, and microarousals interact to influence memory consolidation processes.

Of note, other studies using up-PTAS to enhance motor sequence consolidation have failed to observe behavioral benefits [16, 74]. The variability of stimulation effects between studies, even when conducted in healthy, good-sleeping young adults, remains a major challenge in the field [15]. Identifying optimal stimulation parameters and personalizing PTAS protocols are promising strategies to improve reliability and effect sizes. Our study contributes to ongoing efforts by systematically testing a novel PTAS parameter, the ISI, while implementing state-of-the-art methodological recommendations, including individualized volume calibration [10]. However, ISI represents only one dimension of a broader parameter space. Other critical parameters shown to impact stimulation efficacy include continuous versus intermittent stimulation timing protocols [6, 9], the phase of stimulation relative to the ongoing slow oscillation [11, 62, 75]; and stimulation volume [10, 76].

### Limitations

Although our results provide important insights into a novel parameter in PTAS and hopefully encourage more researchers to perform sleep studies in the home setting, we encountered some limitations that warrant careful consideration.

Firstly, whereas studies conducted in the home setting bear several advantages over classic laboratory studies, translating behavioral tasks to this setting may pose difficulties. In our study, protocol deviations led to the exclusion of two participants. Additionally, there were pre-stimulation differences in learning, evident in the performance score that we needed to account for statistically. Notably, learning trajectories plateaued faster in the long ISI condition than in the short ISI condition, even before the sleep intervention. This ceiling effect was particularly evident in the accuracy component (Supplementary Figure S2A). Given the randomized sequence assignment between conditions, we attribute this behavior to random accuracy fluctuations across trials, whose influence could have been reduced by increasing the sample size. The ceiling effect could have been mitigated by increasing task difficulty, for instance extending the sequence length or having participants use their non-dominant hand, or by dynamically adapting the task difficulty to the individual skill level. We recommend that future studies invest in designing well-standardized, automated tasks for various memory domains with built-in adherence checks while leveraging larger samples to counterbalance some of the variability inherent in studying behavior in the home setting.

Conducting research in the home setting offers a timely and valuable addition to traditional laboratory studies by enhancing ecological validity and scalability and supporting the development of clinically transferable protocols. However, it limited us to EEG data from only one frontal channel and thus precluded the detection of differences in slow oscillation-spindle coupling in task-relevant areas. The limited number of channels also hindered us from pursuing a spindle quantification approach due to small event numbers when detected with the *DETOKS* algorithm, which is, like most automatic spindle detectors, optimized for central channels [45, 77, 78]. Additionally, the headband included over-ear headphones for participant comfort, which bear the risk of applying varying levels of pressure to the ears depending on the sleeping position. This may have introduced additional variability in the perceived sound pressure level and the magnitude of stimulus-evoked responses. Therefore, our study requires validation in the lab setting to better elucidate the mechanisms that contributed to the effects observed.

Secondly, we focused on comparing two active conditions and omitted a stimulation-free sham night to streamline the protocol. Thus, we were limited to comparisons between protocols or within-night ON–OFF windows. Although OFF windows might have been affected by activity from the previous ON-window, potentially confounding ON–OFF contrasts, our findings align with the stimulation responses reported in other studies [5, 20, 62–64]. We cannot entirely exclude possible negative impacts of stimulation on sleep architecture, but our participants’ sleep was within the normal range [79] and thus consistent with other studies that have shown no effects of stimulation on sleep architecture [11, 16]. Furthermore, it remains unclear whether the short ISI condition failed to enhance task consolidation or actively disrupted it, because both scenarios could have led to a significant difference between the short ISI and long ISI protocols. Supporting the first interpretation, previous PTAS studies have reported no effects on memory rather than detrimental effects [16, 74].

Thirdly, current K-complex detectors, including *DETOKS*, still offer limited precision and recall when considering human labels as the ground truth [45]. For instance, *DETOKS* tended to overestimate K-complex densities in N2 sleep compared to previous reports [80]. However, because detection bias is unlikely to differ across ISI bins, we expect the trajectory of stimulus-associated K-complexes to be representative. We refrained from introducing more stringent criteria on duration, amplitude, or slope to maximize the number of K-complexes detected and thus maximize statistical power. We would argue that even human labels, which are driven by morphological features, might not be the optimal ground truth, because K-complexes may change their morphology with increasing sleep depth [54]. Consequently, morphologically atypical K-complexes might be captured by data-driven algorithms such as *DETOKS* but not by the human eye. Future improvements in detection approaches may circumvent these issues and improve detection accuracy.

## Conclusion

Extending studies that have suggested that a higher number of stimuli does not necessarily enhance PTAS effects, we find that a protocol with long ISIs was more effective in improving performance on a finger-tapping sequence than a protocol with shorter ISIs. PTAS stimuli applied at longer ISIs drove K-complexes and spindles more efficiently than PTAS stimuli with short ISIs. The ability to evoke these responses more strongly may have advantages that extend beyond overnight memory consolidation. For example, K-complexes have been reported to regulate cardiovascular responses [81–84] and positively affect heart function [76]. In a related context, K-complexes could also drive waste clearance through the glymphatic system via neurovascular coupling [85]. Importantly, our study shows that the long ISI PTAS protocol can be implemented effectively in the home setting, thus providing a framework for testing its broader application in longitudinal frameworks. To establish the robustness and translational relevance of our findings, future research will need to employ larger samples and evaluate stimulation efficacy across diverse populations and memory domains. In particular, testing PTAS in clinical groups characterized by poor or disrupted sleep will be critical to assessing its clinical utility and generalizability.

## Supplementary Material

Author_Responses_to_Reviewers_SleepAdvances_responsesonly_zpaf073

Supplemental_Material_SleepAdvances_Kasties_et_al_2025_zpaf073

## Data Availability

All data supporting the findings of this study are securely stored at servers of the University Children’s Hospital Zurich. Access and availability will be provided upon a material transfer agreement and after approval by the local Ethics Committee of the Canton of Zurich. Analysis code can be shared upon request.
