# Supplementary material for: Optimizing automated phase-targeted auditory stimulation protocols for procedural memory consolidation during sleep in a home setting
Source: Sleep Adv. 2025 Oct 17;6(4):zpaf073. doi: 10.1093/sleepadvances/zpaf073 (PMC12624865; doi:10.1093/sleepadvances/zpaf073)
Supplement: Supplemental_Material_SleepAdvances_Kasties_et_al_2025_zpaf073 [file supplemental_material_sleepadvances_kasties_et_al_2025_zpaf073.pdf]

Optimizing Automated Phase-Targeted Auditory Stimulation Protocols for Procedural Memory Consolidation during Sleep in a Home Setting

Vanessa Kasties[1,2,3], Nicole Meier[1], Nora-Hjördis Moser[2,3,4], Renske Sassenburg[4], Walter Karlen[5], Maria Laura Ferster[1], Sara Fattinger[1,2], Angelina Maric[2,4], Reto Huber[1,2,6]

1: Child Development Centre, University Children's Hospital Zurich, Zurich, Switzerland

2: Center of Competence Sleep & Health Zurich, University of Zurich, Zurich, Switzerland

3: Neuroscience Center Zurich (ZNZ), University of Zurich and ETH Zurich, Zurich, Switzerland

4: Department of Neurology, University Hospital Zurich (USZ), Zurich, Switzerland

5: Institute of Biomedical Engineering, University of Ulm, Ulm, Germany

6: Department of Child and Adolescent Psychiatry, Psychiatric Hospital, University of Zurich, Zurich, Switzerland

Corresponding author:

Prof. Dr. Reto Huber

University Children`s Hospital Zurich – Eleonore Foundation

Lenggstrasse 30

CH – 8008 Zurich

Switzerland

reto.huber@kispi.uzh.ch

Supplemental Material for Manuscript "Optimizing Automated Phase-Targeted Auditory Stimulation Protocols for Procedural Memory Consolidation during Sleep in a Home Setting"

Supplemental Analyses

Quantification of arousals

To assess whether the two stimulation protocols exhibited differential effects on arousability, we quantified arousals according to AASM definitions[1] using an established algorithm[2,3]. This algorithm was provided with the EEG, EMG, and hypnogram to detect arousals based on increases in the alpha and/or beta activity above a relative threshold, and EMG activation. Signals were filtered forward and backward with 3rd-order Butterworth digital IIR filters with the recommended frequency cutoffs[1]. The EMG quality in our recordings was visually validated. In case of signal loss, the EMG signal was reconstructed from the EOG channels by filtering the signal in the EMG range (10–100 Hz). The results of this analysis are reported in **Table S2**.

Analysis of slow-wave activity response

Average SWA in the low (0.75–1.25 Hz) and adjacent (1.25–4 Hz) frequency bands were calculated separately in ON and OFF windows, and the absolute difference between ON and OFF was obtained as a marker of stimulation response[4–6]. To this end, we selected only ON windows in scored NREM sleep (stages N2 and N3) that contained at least one stimulus and their consecutive OFF windows. SWA was calculated with MATLAB's *pwelch* function for each 6 s window, using 4 s Hanning windows with 50% overlap, and then averaged separately across all ON and OFF windows. The results of this analysis are reported in **Figure S3**.

Control analyses for behavioral effects

To account for pre-stimulation differences in learning with regard to the performance score, we fitted a robust linear mixed-effects model (using the *robustlmm* package in R[7]) predicting performance at retrieval from fixed effects of condition, plateau performance, and their interaction. Random intercepts were included to account for between-subject variance. Plateau

performance scores were mean-centered to ensure that the model intercept is estimated at the average plateau performance instead of at zero, which is implausible given the composition of the score. With this centering, the condition coefficient represents the expected change in retrieval performance for a shift in condition, holding plateau performance at its grand average. The results are summarized in **Table S3**.

To control for potential order effects (i.e., whether a particular stimulation protocol was presented in night 1 or night 2) in comparisons of overnight changes in FTT performance metrics between the stimulation conditions, we fitted robust linear mixed-effects models that included random intercepts for participants and fixed effects for condition, night, and night×condition interaction. See **Tables S4 – S5** for detailed summaries of these models.

To exclude the possibility that the distribution of stimuli across different sleep stages would impact the results, we tested for differences in the relative number of stimuli in N3 (% stimuli in N3 relative to all NREM). We found no significant difference between conditions (long ISI: *Med* = 90.04%, *IQR* = 7.16%, short ISI: *Med* = 91.06%, *IQR* = 15.10%, long ISI>short ISI: g = 0.20, p = 0.980, Wilcoxon signed-rank test).

References

1. Berry RB, Brooks R, Gamaldo CE, et al. *The AASM Manual for the Scoring of Sleep and Associated Events: Rules, Terminology and Technical Specifications*. Version 2.4. American Academy of Sleep Medicine, Darien, IL; 2017.
2. Alvarez-Estevez D, Fernández-Varela I. Large-scale validation of an automatic EEG arousal detection algorithm using different heterogeneous databases. *Sleep Med*. 2019;57:6-14. doi:10.1016/j.sleep.2019.01.025
3. Fernández-Varela I, Alvarez-Estevez D, Hernández-Pereira E, Moret-Bonillo V. A simple and robust method for the automatic scoring of EEG arousals in polysomnographic recordings. *Comput Biol Med*. 2017;87:77-86. doi:10.1016/j.compbiomed.2017.05.011
4. Lustenberger C, Ferster ML, Huwiler S, et al. Auditory deep sleep stimulation in older adults at home: a randomized crossover trial. *Communications Medicine*. 2022;2(1). doi:10.1038/s43856-022-00096-6
5. Papalambros NA, Santostasi G, Malkani RG, et al. Acoustic enhancement of sleep slow oscillations and concomitant memory improvement in older adults. *Front Hum Neurosci*. 2017;11. doi:10.3389/fnhum.2017.00109

6. Grimaldi D, Papalambros NA, Reid KJ, et al. Strengthening sleep–autonomic interaction via acoustic enhancement of slow oscillations. *Sleep*. 2019;42(5). doi:10.1093/sleep/zsz036

7. Koller M. Robustlmm: An R package for Robust estimation of linear Mixed-Effects models. *J Stat Softw*. 2016;75(1). doi:10.18637/jss.v075.i06

Supplemental Figures

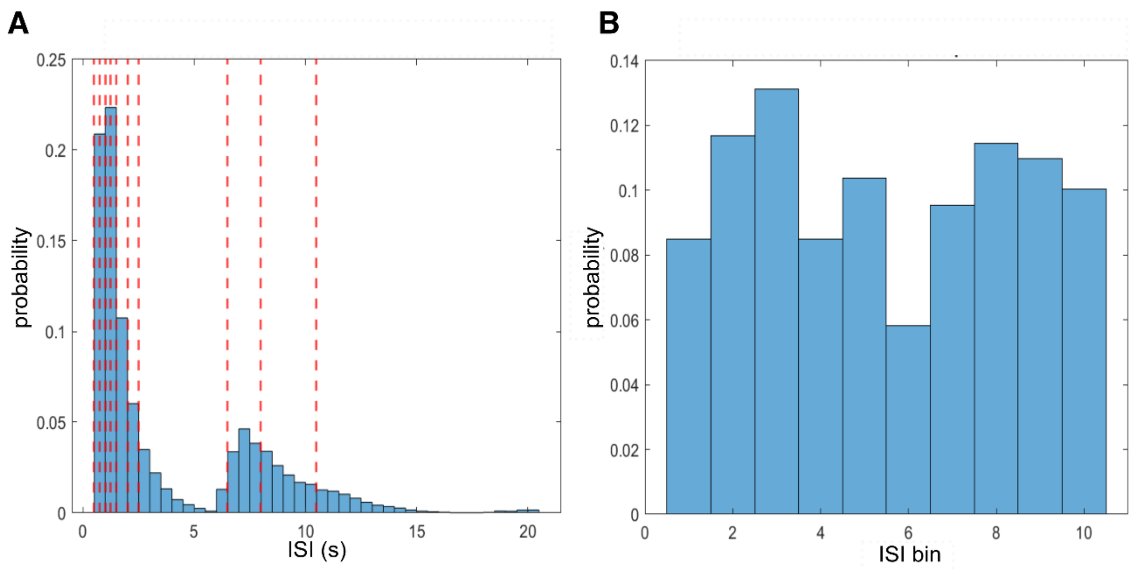

Figure S1: **Data-driven definition of ISI bins. (a)** Histogram of interstimulus intervals across the sample. Red dashed lines represent bin edges defined based on nine quantiles rounded down to the nearest 0.5 s, resulting in edges at 0.5 s (duplicate), 1 s (duplicate), 1.5 s, 2.5 s, 6.5 s, 8 s, and 10.5 s). The range between 0.5 and 2.5s was then subdivided into smaller steps for a more fine-grained parcellation, adding edges at 0.75 s, 1.25 s, and 2.0 s. **(b)** Histogram of ISI bin distribution across the sample.

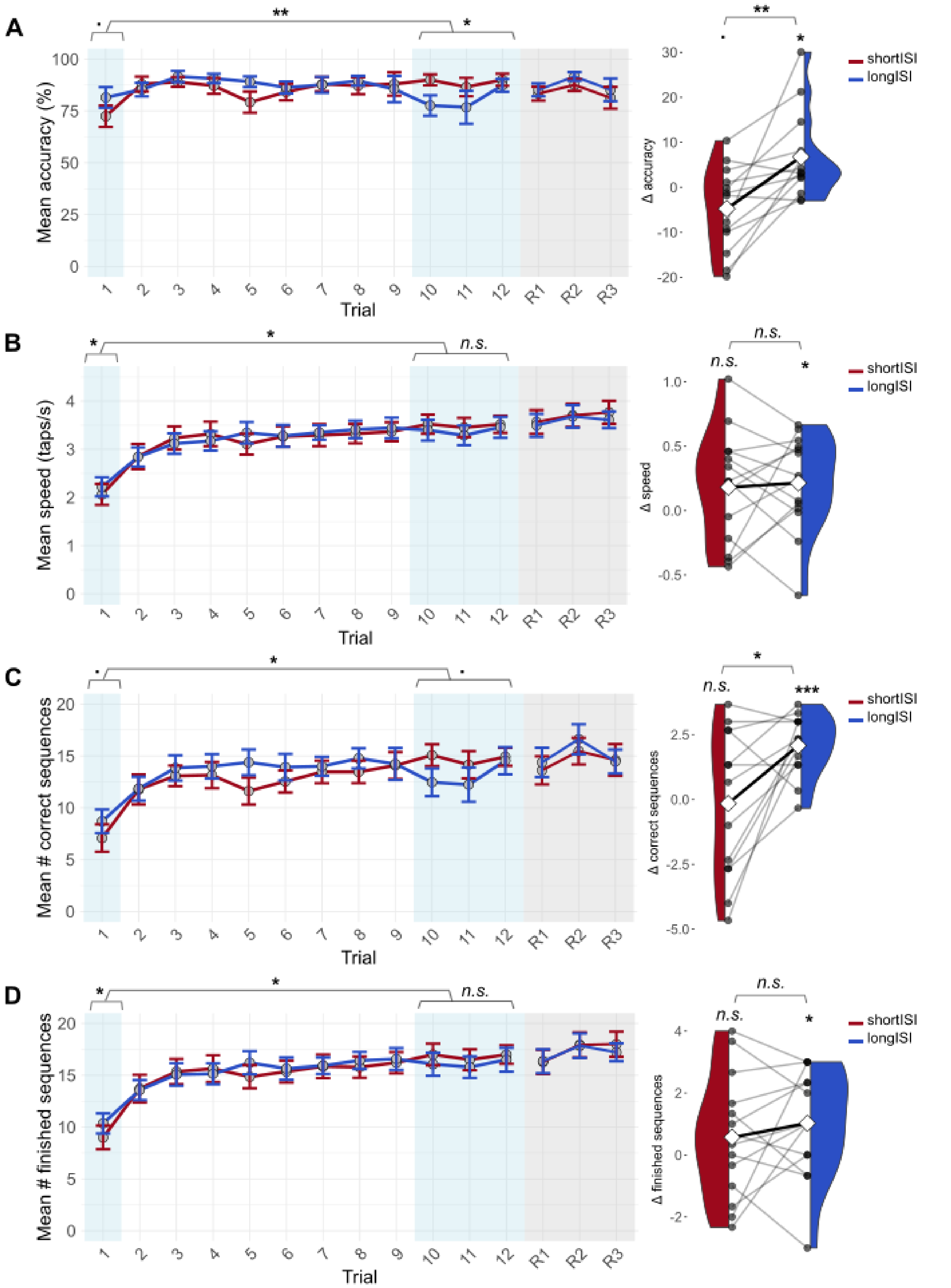

Figure S2: Comparison of learning trajectories and overnight change of alternative FTT performance metrics. Trajectories across learning (1 — 12) and retrieval (R1 — R3) trials and comparisons of overnight change of (a) accuracy, (b) speed, (c) number of correct sequences, and (d) number of finished sequences between short and long ISI conditions. Grey circles indicate means across participants, and error bars indicate standard errors of the mean. Asterisks represent significant differences between conditions in baseline, learning rate, plateau, and overnight gains with *** $p < 0.001$, ** $p < 0.01$, *$p < 0.05$, n.s. not significant; paired two-tailed Student's *t* test. Asterisks above violin plots

mark significant differences in the performance metric at retrieval versus at plateau, calculated separately for each condition.

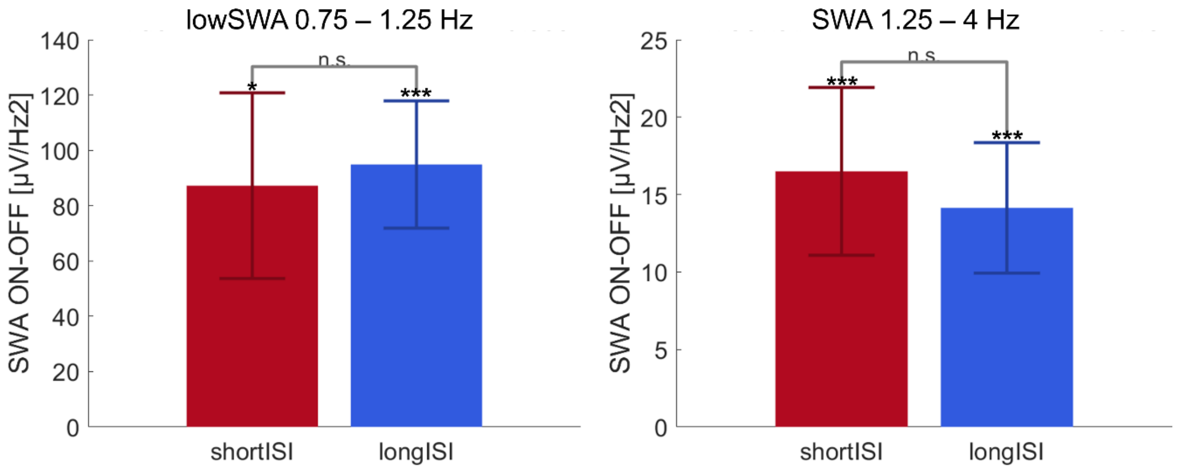

Figure S3: Comparison of slow wave activity change (ON – OFF) between stimulation conditions. Both protocols showed significantly increased activity in the low SWA band (0.75–1.25 Hz; one-tailed one-sample *t* test) and the adjacent SWA range (1.25–4 Hz; one-tailed Wilcoxon signed-rank test), with no significant differences between protocols (paired two-tailed Student's *t* test). Asterisks represent *p* values with *** $p < 0.001$, ** $p < 0.01$, * $p < 0.05$, n.s. not significant.

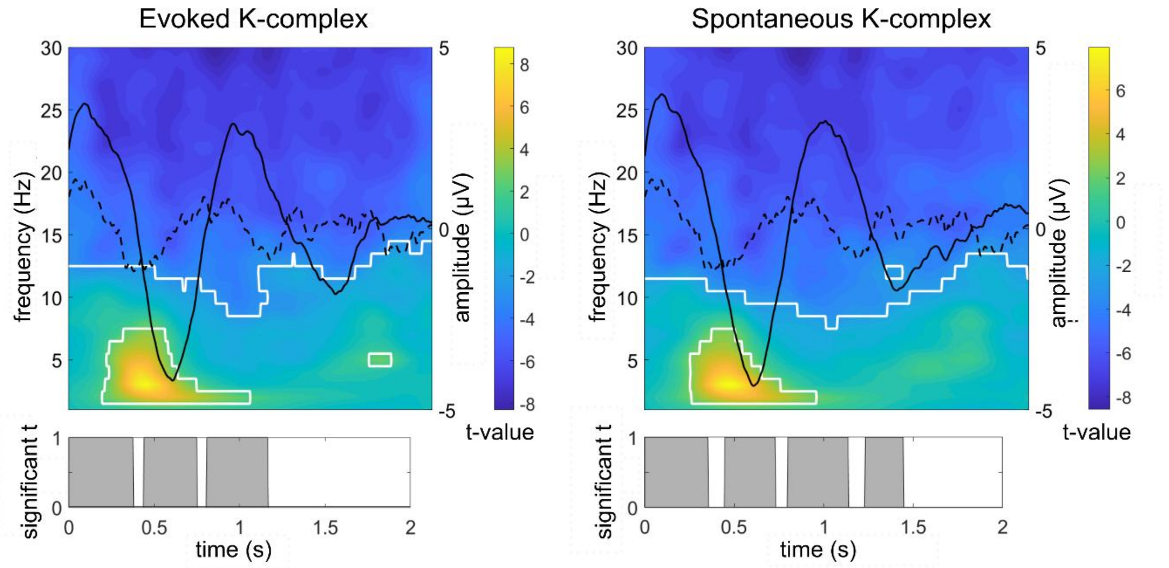

Figure S4: Evoked (ON-window) and spontaneous (OFF-window) K-complexes detected by *DETOKS*. Solid black lines represent average waveform across 2 s epochs locked to the starting point of detected K-complexes associated with ON-stimuli and sham stimuli in OFF windows. Dashed black lines represent average waveform across random 2 s NREM epochs not labeled as K-complexes. The bottom plot indicates areas where the difference between K-complex waveform and random NREM epoch waveform was significant after permutation-based cluster correction (paired two-tailed Student's *t* tests, $\alpha = 0.05$). The *t* maps relate to power in the time-frequency space, contrasting K-complex epochs and random NREM epochs. White margins indicate significant clusters after permutation-based cluster correction (paired two-tailed Student's *t* tests, $\alpha = 0.05$).

Supplemental Tables

Table S1: Detailed comparison of NREM sleep stage 1 amount within the stimulated† and unstimulated†† part of the night (*N* = 16).

	Short ISI (mean ± SD)	Long ISI (mean ± SD)	Mean Difference	95% CI	*g*	*p*
N1 stimulated [min]	8 ± 4.8	10 ± 8.5	2.0	[-2.6, 6.6]	0.27	0.365
N1 unstimulated [min]	14 ± 8.4	19 ± 8.1	5.0	[0.1, 9.8]	**0.57**	**0.044**
N1 stimulated [%]	4.1 ± 2.51	4.9 ± 3.91	0.76	[-1.34, 2.86]	0.22	0.452
N1 unstimulated [%]	5.0 ± 2.63	6.8 ± 2.94	1.78	[0.16, 3.40]	**0.61**	**0.033**
N1 [%]	4.7 ± 2.05	6.0± 2.87	1.32	[0.11; 2.53]	0.48	**0.035**

Mean differences refer to the contrast longISI > shortISI. The reported *p* values were obtained by paired Student's *t* tests (*N* = 16), and the effect size is reported as Hedge's *g*. Significant comparisons are marked in bold.
† Stimulated part of the night: 2.5 h after the first stimulus. †† Unstimulated part of the night: from 2.5 h after the first stimulus to the end of the recording.

Table S2: Comparison of automatically detected arousals between stimulation conditions (*N* = 16).

	Short ISI (mean ± SD)	Long ISI (mean ± SD)	Mean Difference	95% CI	*g*	*p*
Arousal number	76 ± 29.8	68 ± 23.8	-8	[-19.5, 4.0]	0.27	0.180
Arousal density [1/min]	0.16 ± 0.06	0.14 ±0.05	-0.02	[-0.05, 0.01]	0.31	0.156
Mean arousal duration [s]	7.94 ± 1.32	7.98 ± 1.15	0.04	[-0.54, 0.62]	-0.03	0.893
Total time spent in arousal [min]	9.8 ± 3.48	9.0 ± 3.07	-0.8	[-2.0, 0.4]	0.23	0.162
Relative time spent in arousal [%]	2.1 ± 0.72	1.9± 0.71	-0.2	[-0.49, 0.07]	0.28	0.135

Mean differences refer to the contrast longISI > shortISI. The reported *p* values were obtained by paired Student's *t* tests (*N* = 16) and the effect size is reported as Hedge's *g*.

Table S3: Fixed effects of robust linear mixed-effects model (rLMM) including performance at plateau and its interaction with condition as covariates to predict the performance score at retrieval (*N* = 13).

	Estimate	Standard Error	*t* value	Approximated *p* value
intercept	292.29	15.00	**19.491**	**< 0.001**
condition	39.95	17.95	**2.225**	**0.037**
plateau (mean-centered)	0.98	0.21	**4.721**	**< 0.001**
plateau×condition	-0.07	0.24	-0.287	0.777

Significant comparisons are marked in bold.

Table S4: Fixed effects of rLMM including order (night 1 or 2) effects as covariates to predict the overnight change in performance score (*N* = 13).

	Estimate	Standard Error	*t* value	Approximated *p* value
intercept	2.00	14.68	0.136	0.893
condition	42.65	17.18	**2.483**	**0.021**
night	-9.96	20.76	-0.480	0.636
night×condition	0.39	33.68	0.012	0.991

Significant effects are marked in bold.

Table S5: Fixed effects of rLMM including order effects as covariates to predict the overnight change in tapping variability (*N* = 14).

	Estimate	*Standard Error*	*T-value*	*Approximated p-value*
intercept	13.67	15.28	0.895	0.380
condition	-32.36	15.19	**-2.130**	**0.044**
night	-10.92	21.60	-0.506	0.617
night×condition	-2.47	37.48	-0.066	0.948

Significant comparisons are marked in bold.

Table S6: Comparison of detected K-complexes and stimuli associated with K-complexes between the stimulation conditions (*N* = 16).

	shortISI (mean ± SD)	shortISI (min - max)	longISI (mean ± SD)	longISI (min - max)	Mean Difference	95% CI	*p*	*g*
NREM sleep K-complex density [1/min]	9.8 ± 2.11	5.9 – 12.8	10.0 ± 1.86	5.7 – 12.4	0.3	[-0.57; 1.07]	0.53	0.12
N2 sleep K-complex density [1/min]	7.0 ± 2.28	3.6 – 10.7	6.8 ± 1.95	3.2 – 10.0	-0.1	[-1.01; 0.75]	0.75	-0.06
N3 sleep K-complex density [1/min]	16.1 ± 1.81	12.6 – 18.5	16.6 ± 1.82	13.1 – 19.1	0.5	[-0.40; 1.45]	0.25	0.27
NREM sleep K-complex density 1st half [1/min]	11.8 ± 1.72	8.3 – 15.0	12.0 ± 1.97	8.6 – 14.8	0.2	[-0.53; 0.89]	0.59	0.09
N2 sleep K-complex density 1st half [1/min]	8.0 ± 1.71	4.8 – 11.9	7.3 ± 2.20	3.2 – 11.7	-0.6	[-1.50; 0.24]	0.14	-0.30
N3 sleep K-complex density 1st half [1/min]	15.6 ± 1.87	12.5 – 18.5	16.6 ± 1.98	13.1 – 19.4	1.0	[0.23; 1.76]	**0.01**	**0.49**
NREM sleep K-complex density 2nd half [1/min]	8.2 ± 3.17	3.1 – 12.6	8.3 ± 2.57	3.4 – 11.9	0.2	[-1.22; 1.52]	0.82	0.05
N2 sleep K-complex density 2nd half [1/min]	6.5 ± 2.48	3.0 – 11.3	6.7 ± 2.11	3.1 – 9.6	0.2	[-0.82; 1.19]	0.70	0.08
N3 sleep K-complex density 2nd half [1/min]	14.8 ± 6.35	0.0 – 21.3	15.6 ± 4.97	0.0 – 20.7	0.8	[-1.62; 3.16]	0.50	0.12
Stimuli with K-complex	299 ± 131.5	110 – 512	204 ± 90.8	46 – 369	-94.3	[-157.3; -31.4]	**< 0.01**	**-0.77**

Stimuli without K-complex	800 ± 390.3	260 – 1516	434 ± 181.1	140 – 766	-365.9	[-530.2; -201.5]	**< 0.001**	**-0.96**
Proportion K-complex evoking stimuli [%]	27.9 ± 5.27	19.6 – 38.2	31.8 ± 5.24	23.3 – 41.2	3.9	[1.60; 6.74]	**0.01**	**0.71**

Mean differences refer to the contrast longISI > shortISI. The reported *p* values were obtained by paired Student's *t* tests (*N* = 16), and effect size is reported as Hedge's *g*. Significant comparisons are marked in bold. Abbreviations: N2 – NREM sleep stage 2, N3 – NREM sleep stage 3.
