# Supplementary material for: Optimizing automated phase-targeted auditory stimulation protocols for procedural memory consolidation during sleep in a home setting
Source: Sleep Adv. 2025 Oct 17;6(4):zpaf073. doi: 10.1093/sleepadvances/zpaf073 (PMC12624865; doi:10.1093/sleepadvances/zpaf073)
Supplement: Author_Responses_to_Reviewers_SleepAdvances_responsesonly_zpaf073 [file author_responses_to_reviewers_sleepadvances_responsesonly_zpaf073.docx]

**Reviewer Comments with Author Responses related to the Manuscript SLEEPADV-2025-019: *“Optimizing Automated Phase-Targeted Auditory Stimulation Protocols for Procedural Memory Consolidation during Sleep in a Home Setting”***

Changes resulting from language editing are marked in blue, whereas changes addressing the reviewer’s comments are marked in red in the revised manuscript.

***Associate Editor***

*Comments to the Author*

1. *Two reviewers have provided critiques of your manuscript about this interesting study. In addition to the clarifications that were requested in their reviews, both critiques raised the finding of pre-stimulation performance differences in the ISI conditions.*

Thank you for summarizing the two reviewers’ comments about this point. As described in detail in the responses to Reviewer 1, we have included an additional control model to account for pre-stimulation differences in the performance score in the *Supplemental Material*. According to the model estimates, pre-stimulation performance differences (the interaction of plateau performance and condition) had no significant influence on post-stimulation retrieval performance scores, and the condition effect remained significant.

2. *As reviewer 1 also mentioned in their first point, the method describes a word pair task was also completed. Including results on that task (positive or negative findings) would strengthen the paper.*

It is true that the original version of the manuscript described a word-pair task as part of our test battery. We had run a pilot on the first six participants of this study to test our novel mobile application that implemented an automated version of this task. Data collection was discontinued after the first six participants due to quality concerns arising from the unsupervised setting in which the task was performed. Due to the lack of reliable data quality and an insufficient number of participants, we are unable to report any analyses of the word-pair task in the manuscript. Instead, we focus on the finger-tapping task, which offered a more standardized and structured procedure. However, we have clarified the inclusion of the word-pair task in the study design in the *Methods* (p.8) for transparency.

3. *Reviewer 2 also asked about some additional finger tapping performance metrics that might be useful to include.*

As suggested by Reviewer 2, we have included analyses related to other finger-tapping performance metrics (as in ^1–4^) in the *Supplemental Material*. These metrics all point to more favorable performance outcomes in the long ISI compared to the short ISI condition.

***Reviewer:1*** *Comments to the Author*

*The paper presents a study examining effects of interstimulus interval during at-home up-phase targeted auditory stimulation in healthy adults on procedural memory consolidation using a finger-tapping task. Auditory stimulation has mixed effects, potentially due to differences in the stimulation protocol. Therefore, this study seeks to determine whether ISI is an important parameter to optimize the effectiveness of auditory stimulation and memory consolidation.*

1. *I would have liked to see different kinds of overnight memory tasks used – such as also a word pair task – which has been commonly used in other auditory stimulation studies. Especially since different aspects of sleep might be more important for different kinds of memories. In fact, there is a brief mention in the experimental protocol that a word pair task was given but is not mentioned anywhere else. Did participants also complete a word pair task or was this a typo?*

It is true that we have piloted a newly designed mobile application that featured an automated version of the word-pair task (adapted from ^5^) within this study. However, we stopped presenting the task after the first six participants due to quality concerns; the application required written responses, but in the unsupervised setting, we could not ensure robustness to typos or prevent the use of auxiliary aids. To acknowledge the task in the study design, we include a statement in the *Methods* (p.8):

“As part of a piloting procedure, an unsupervised word-pair task was presented to the first six participants between the FTT and PVT. Data from this task was not included in the analyses.”

2. *While the authors do mention that the sample size was small, I would still like this point to be highlighted more in the limitations, particularly given that it is acknowledged that effects of auditory stimulation tend to be small so it is unclear why an n of 18 was chosen.*

We fully agree that larger samples will be required to prove the robustness of our findings, which we now highlight in the *Conclusion* (p.28):

“To establish the robustness and translational relevance of our findings, future research will need to employ larger samples and evaluate stimulation efficacy across diverse populations and memory domains.”

As this is the first study that manipulates ISI in PTAS systematically, we oriented our sample size at the average sample size reported in previous studies (N = 16^6^). We still believe the findings will be of interest to the research community by helping to establish novel PTAS parameters.

3. *I would be interested to hear the authors thoughts, and some reference in the discussion, on the generalizability and clinical utility of optimizing the auditory stimulation protocol in healthy young adults with good sleep, as opposed to in individuals with poor sleep.*

We thank the reviewer for this important point. We agree that ultimately, the clinical utility of such optimization lies in its translation to populations with poor or disrupted sleep. To acknowledge this point, we have included this statement in the *Conclusion* (p.29):

“In particular, testing PTAS in clinical groups characterized by poor or disrupted sleep will be critical for assessing its clinical utility and generalizability.”

However, we view the optimization and personalization of stimulation protocols in healthy individuals as a necessary first step, given the considerable variability in stimulation effects across studies, even when conducted in healthy young adults^6^. Working within a more stable and better-understood sleep architecture allows us to isolate and evaluate the influence of specific parameters before introducing the additional complexity of clinical populations. To give more credit to this reasoning, we included the following paragraph in the *Discussion* (p.26):

“Of note, other studies using up-PTAS to enhance motor sequence consolidation have failed to observe behavioral benefits^7,8^. The variability of stimulation effects between studies, even when conducted in healthy, good-sleeping young adults, remains a major challenge in the field^6^. Identifying optimal stimulation parameters and personalizing PTAS protocols are promising strategies to improve reliability and effect sizes. Our study contributes to ongoing efforts by systematically testing a novel PTAS parameter, the ISI, while implementing state-of-the-art methodological recommendations, including individualized volume calibration^9^. However, ISI represents only one dimension of a broader parameter space. Other critical parameters shown to impact stimulation efficacy include continuous versus intermittent stimulation timing protocols^10,11^, the phase of stimulation relative to the ongoing slow oscillation^12–14^, and stimulation volume^9,15^.

We have also clarified our contribution in the *Abstract* and *Introduction* of the revised manuscript:

Abstract: “Our study contributes to ongoing efforts in characterizing parameter choices in PTAS and compares two up-PTAS protocols with systematic variations of the interstimulus intervals (ISIs) and their effect on the consolidation of a finger-tapping sequence.”

Introduction (p.6): “Our results confirm the feasibility of PTAS studies in the home setting and provide the first experimental evidence on how ISI length impacts PTAS efficacy and behavior.”

4. *The authors mention that the FTT performance results need to be interpreted with caution because learning trajectories pre-stimulation were already different between ISI conditions. Why not control for pre-stimulation performance in these analyses to strengthen the claims about post-stimulation performance?*

Thank you for your suggestion. To address potential confounds from pre-stimulation differences in plateau performance, we followed an approach from a previous study^16^ and fitted a robust linear mixed-effects model predicting performance at retrieval from fixed effects of condition, plateau performance, and their interaction. The model is described in more detail in *Supplemental Analyses.* We refer to these additional analyses in the *Results* (p.18):

“Given that a pre-stimulation difference in performance scores was observed between conditions, we fitted a separate robust linear mixed-effects model to control for plateau performance and its interaction with condition (**Supplemental Analyses**). The dependent variable in this case was the performance score at retrieval. As expected, the main effect of plateau performance was significant (β = 0.98, *t* = 4.721**,** *p* < 0.001), indicating that retrieval performance increased with the performance level achieved during prior learning. The model indicated no significant contribution of the plateau$\times$condition interaction to retrieval scores, whereas the condition effect remained significant (β = 39.95, *t* = 2.225**,** *p* = 0.037; **Table S3**).”

Furthermore, we could strengthen some claims in the *Discussion*. As a consequence, we have integrated the findings related to performance scores in the following paragraph (p.24):

“Our second hypothesis predicted that the long ISI up-PTAS protocol would drive overnight performance change more efficiently than the short ISI protocol. In agreement with a study rejecting the idea of a linear relationship between stimulus number and PTAS effects on sleep-dependent memory consolidation^17^ the long ISI protocol proved more effective in increasing performance scores and decreasing tapping variability overnight despite involving significantly fewer stimuli. These overnight performance improvements, so-called “offline gains”, were previously observed for the performance score as a consequence of sleep^18^. The overnight decrease in tapping variability observed in the long ISI condition relative to the short ISI condition could indicate more fluent and stable motor performance. This finding aligns with previous observations, according to which sleep selectively improved the speed of key press transitions that were the slowest before sleep^19^.”

We have removed the paragraph on pre-stimulation learning differences (previously p.25), as these differences have already been accounted for by modeling. Eliminating this passage avoids redundant relativizing statements and allows the discussion to focus more directly on the core findings, thereby strengthening our claims regarding performance scores, as suggested. However, we continue to discuss ceiling effects and the pre-stimulation differences in the *Limitations* (p.26):

“Firstly, whereas studies conducted in the home setting bear several advantages over classic laboratory studies, translating behavioral tasks to this setting may pose difficulties. In our study, protocol deviations led to the exclusion of two participants. Additionally, there were pre-stimulation differences in learning, evident in the performance score, that we needed to account for statistically. Notably, learning trajectories plateaued faster in the long ISI condition than in the short ISI condition, even before the sleep intervention. This ceiling effect was particularly evident in the accuracy component (**Figure S2A**). Given the randomized sequence assignment between conditions, we attribute this behavior to random accuracy fluctuations across trials, whose influence could have been reduced by increasing the sample size. The ceiling effect could have been mitigated by increasing task difficulty, for instance extending the sequence length or having participants use their non-dominant hand, or by dynamically adapting the task difficulty to the individual skill level. We recommend that future studies invest in designing well-standardized, automated tasks for various memory domains with built-in adherence checks while leveraging larger samples to counterbalance some of the variability inherent in studying behavior in the home setting.”

We continue to report the paired t-test as the main analysis, as it is intuitive for readers to understand and removes between-participant variability in plateau performance within the analysis, which in our small sample produces smaller standard errors than a conditional mixed model.

4. *There is not much discussion on why the long ISI condition experienced significantly more %N1 sleep than the short ISI condition. Could the authors speculate on why this occurred?*

Excellent question! We examined this issue more thoroughly and investigated where exactly the difference in N1 sleep occurred. To this end, we divided each night into two time periods: one *with* stimulation (from the beginning of the recording until 2.5 hours after the first stimulus) and one *without* stimulation (from the point where the stimulation period ended to the end of the night). We found that the difference in N1 sleep, both in absolute and relative terms, was only significant in the second, unstimulated part of the night.

Considering that N1 is often staged after arousals according to the AASM scoring guidelines, we wondered whether a difference in arousal density could explain the difference in N1 sleep. Using a validated detection algorithm to identify arousals according to AASM criteria^20,21^, we found no significant differences between long ISI and short ISI nights in any of the arousal metrics we defined. Taken together, we do not believe that the difference in N1 is directly related to the stimulation but rather a result of night-to-night variability. We now report these additional analyses in the *Supplemental Material* and summarize the findings in the *Results* section (p.16).

5. *There is a concern, which the authors partly acknowledge, about the ability to actually detect sleep-based changes caused by auditory stimulation (and therefore long vs short ISI condition differences), such as slow oscillation-spindle coupling. There are advantages to testing in a home environment using a headband device. However, I wonder if this was the best approach given the goals of the current study (optimizing the effectiveness of automated stimulation protocols) which might have been served better with in-lab PSG.*

This manuscript describes one of a series of studies being conducted by our research group, both inside and outside of the lab, for the purpose of improving phase-targeted auditory stimulation protocols (see, for example, our recent preprint of a lab-based study on this topic, https://doi.org/10.1101/2025.03.05.641406). We are happy to refer to other studies of this series in our revised manuscript if they get published in the meantime. We agree that the current study needs to be validated in a laboratory setting, especially to strengthen the mechanistic aspects and to confirm the robustness of the behavioral findings. However, we believe that conducting research in the home setting offers a timely and valuable addition to traditional lab studies, providing greater ecological validity and scalability, and supporting the development of clinically transferable protocols.

We made the following extensions to the *Limitations* (p.27) to recognize this point:

“Conducting research in the home setting offers a timely and valuable addition to traditional laboratory studies by enhancing ecological validity and scalability and supporting the development of clinically transferable protocols. However, it limited us to EEG data from only one frontal channel, and thus precluded the detection of differences in slow oscillation-spindle coupling in task-relevant areas. […]. Therefore, our study requires validation in the lab setting to better elucidate the mechanisms that contributed to the observed effects.”

**Reviewer: 2**

*Comments to the Author*

*In this article, the authors investigated the effects of auditory stimulation on performance in a finger tapping task using a mobile device and app-based behavioral assessments conducted in a home setting. They found that the longer ISI condition was associated with reduced tapping variability. The study is potentially interesting in demonstrating the feasibility of app-based assessments in home environments. However, there are a large number of limitations in the experimental design, data analysis, and interpretation of the results that need to be addressed.*

1. *Statements regarding memory consolidation and the effects of longer ISI should be toned down, as the current study design does not allow for causal inferences, and the findings are not sufficiently robust.*

Thank you for this important point. We agree that, while our experimental design allows for within-subject comparisons across stimulation conditions, the strength of causal inference is limited by the small sample size, and the findings may not generalize beyond the specific memory task used. In response, we have revised the manuscript to tone down interpretive language regarding memory consolidation (e.g., by removing parts of the *Discussion* that put too much focus on memory consolidation mechanisms; deletions highlighted in the *trackchange* version) and have added clarification that the findings pertain specifically to the observed task performance (e.g., by replacing “procedural memory consolidation” by more task-specific terms like “overnight consolidation of a finger-tapping sequence” or “overnight changes in finger-tapping performance”). Where applicable, we also toned down certain statements that relate to the interpretation of our results (e.g. by replacing being “predicted” by being “positively correlated with” or “associated with”) or used hedging language in more generalized statements (e.g., “up-PTAS *may* be optimized for overnight procedural memory consolidation”). We also include a qualifying statement in the *Conclusion* (p.28-29):

“To establish the robustness and translational relevance of our findings, future research will need to employ larger samples and evaluate stimulation efficacy across diverse populations and memory domains. In particular, testing PTAS in clinical groups characterized by poor or disrupted sleep will be critical for assessing its clinical utility and generalizability.“

2. *If I understand correctly, there were already significant differences in performance between conditions before sleep.*

As detailed in the response to Reviewer 1, we have addressed potential confounds related to pre-stimulation differences in performance scores by including an additional control model in the *Supplemental Material* of the revised manuscript. A detailed model description can be found in **Supplemental Analyses***,* for results see **Table S3**. The estimates from this model continue to support our conclusions about the favorable effect of the long ISI stimulation protocol.

3. *Furthermore, I am concerned that the shorter ISI condition may have been associated with lower sleep quality.*

Thank you for raising this concern. Based on the control analyses conducted to date, we found no statistical evidence suggesting that the short ISI condition negatively impacted sleep quality. Specifically, we observed no significant differences between the two conditions in measures of sleep architecture, subjective sleepiness (Karolinska Sleepiness Scale), or response speed on the psychomotor vigilance task (**Table 1**).

Given that sleep quality could be impacted by the presence of arousals, we extended our analyses to include a comparison of arousals between conditions. Using a validated detection algorithm to identify arousals according to AASM criteria^20,21^, we again found no significant differences between long ISI and short ISI nights. We now report this additional analysis in the *Supplemental Material*, as referenced in *Results* (p.16). In summary, these findings indicate that differences in measures of objective and subjective sleep quality are unlikely to explain the difference in finger-tapping performance metrics across stimulation conditions.

4. *The study also does not clearly clarify how K-complexes are related to memory consolidation.*

Thank you for raising this issue. We acknowledge that the interchangeable use of “slow oscillation” and “K-complex” in the manuscript may have caused confusion. In an attempt to disentangle this, we have revised the *Introduction* (p.4-5) as follows:

“Up-PTAS relies on applying short (50 ms) bursts of pink noise at the up-phase of ongoing slow oscillations^12^. These stimuli are thought to induce a rapid and synchronous neuronal depolarization via bottom-up processes — a response that closely resembles the generation of a K-complex^9^. Functionally, this K-complex can be considered equivalent to a stimulus-evoked slow oscillation (< 1 Hz), which is thought to participate in the orchestration of thalamic spindles (oscillatory bursts of 11 – 16 Hz) and hippocampal ripples to the depolarizing up-phase^12^. This precise temporal coordination of sleep oscillations is assumed to be critical for memory consolidation^22,23^. […] A recent study from our laboratory showed that up-phase targeted stimuli played in short succession tend to induce a long SWA plateau, whereas isolated stimuli reliably evoke a K-complex response, irrespective of the targeted phase^5^. This observation agrees with early reports of evoked K-complexes being susceptible to habituation or refractory processes^24–26^. Interestingly, the K-complex response in our study was accompanied by spindles that were preferentially nested in its up-phase. This induced spindle nesting was positively correlated with the overnight retention of previously learned word pairs^20^.”

5. *I wonder whether the authors considered additional performance metrics commonly used in finger tapping tasks, such as the number of correctly typed responses. It was unclear from Figure S3 whether any significant differences existed between conditions.*

Thank you for your suggestion. We have added analyses of additional performance metrics commonly used in finger-tapping tasks to the *Supplemental Material*, referred to in *Results*, p.17. As these metrics show effects consistent with those observed for our original measures (performance and tapping variability), we believe that they strengthen the robustness of our initial findings in favor of the long ISI condition.

Specifically, we now report the following:
– **Accuracy**, defined as the percentage of correct sequences among finished sequences (included in performance measure^2^)
– **Speed**, measured as taps per second (included in performance measure^2^)
– **Number of correct sequences**^1,4^
– **Number of finished sequences**^3,4^

We acknowledge that the original visualization in **Figure S3** (now **Figure S2**) was not very intuitive. To improve clarity, we now present these analyses using the same format as in **Figure 2**, which we believe more clearly illustrates the differences between conditions. See the revised Figure below.

**Figure S2: Comparison of learning trajectories and overnight change of alternative FTT performance metrics.** Trajectories across learning (1—12) and retrieval (R1—R3) trials and comparisons of overnight change of **(a)** accuracy, **(b)** speed, **(c)** number of correct sequences, and **(d)** number of finished sequences between short and long ISI conditions. Gray circles indicate means across participants, and error bars indicate standard errors of the mean. Asterisks represent significant differences between conditions in baseline, learning rate, plateau, and overnight gains with *** *p* < 0.001, ** *p* < 0.01, **p* < 0.05, n.s. not significant; paired two-tailed Student’s *t* test. Asterisks above violin plots mark significant differences in the performance metric at retrieval versus at plateau, calculated separately for each condition.

6. *Was there a significant offline gain (i.e., significantly improved performance after sleep compared to before sleep) in the long ISI condition? This was not clear from Figure 2.*

Thank you for pointing this out. We have added the results related to the pre-post comparisons within conditions to the *Results* section (p.17) and to **Figure 2**.

The corresponding paragraph now reads:

“Performance scores increased significantly more in the long ISI condition than in the short ISI condition (*g* = 0.81*, p* = 0.022*;* long ISI: mean overnight change = 44.12, *p* < 0.001*,* short ISI: mean overnight change = 0.90, *p* = 0.959), after excluding one outlier from the sample (see *Statistics* section for outlier definition; **Figure 2C**). This outlier was excluded from all subsequent analyses related to the performance score. […]. Likewise, overnight tapping variability significantly differed between the long and short ISI conditions (*g* = -0.64*, p* = 0.039), with a decrease observed in the long ISI condition (mean overnight change = -19.09, *p* = 0.146) and a slight increase in the short ISI condition (mean overnight change = 15.81, *p =* 0.310; **Figure 2D**).”

Please find the revised figure below.

**Figure 2:** **Learning and consolidation of the finger-tapping task between long ISI and short ISI conditions. (a)** and **(b)** Trajectories of performance and tapping variability across learning (1—12) and retrieval (R —R3) trials. Gray circles indicate means across participants and error bars indicate standard errors of the mean. Blue-shaded areas represent the baseline and learning plateau, and grey-shaded areas indicate retrieval. Asterisks represent significant differences in baseline, learning rate, and plateau with **p* < 0.05, n.s. not significant; paired two-tailed Student’s *t* test. **(c)** The overnight change in performance was significantly different between short ISI and long ISI conditions (*p* < 0.05, *N* = 13, paired two-tailed Student’s *t* test). One outlier was removed from the comparison for being >3 SDs away from the population mean. **(d)** The overnight change in tapping variability was significantly different between short ISI and long ISI conditions (*p* < 0.05, *N* = 14, paired two-tailed Student’s *t* test). In **(c)** and **(d)**, white diamonds indicate condition means, bold black line indicates mean difference, gray circles represent individual participants, and gray lines represent within-subject differences. Asterisks above violin plots represent significant differences in the performance metric at retrieval versus at plateau (*** *p* < 0.001, n.s. not significant), calculated separately for each condition, while horizontal brackets refer to differences in overnight gains between conditions. **(e)** Overnight change in performance in the FTT by condition and median ISI with linear model fit. Only the main effect of median ISI (β = -216.27, *t* = -3.13, *p* = 0.005) was significant, but not the interaction of condition and median ISI (β = 65.18, *t* = 0.61, *p* = 0.548). Robust percentage bend correlations were not significant in any condition. **(f)** Overnight change in tapping variability in the FTT by condition and median ISI with linear model fit. The interaction of condition and median ISI was significant (β = -285.04, *t* = -2.013, *p* = 0.047). Robust percentage bend correlations indicated a significant association between ISI and overnight change in tapping variability only in the long ISI condition. In **(e)** and **(f)**, solid lines indicate the model prediction, gray shaded areas the 95% CI.

7. *There appear to be large inter-individual differences in sound pressure levels. It is helpful if the authors could clarify the reasons for this.*

Thank you for this observation. As described in the *Methods* section (p.10), the variability in sound pressure levels reflects our individualized approach to setting the stimulation volume. This was based on two criteria: (1) the participant's hearing threshold during wakefulness and (2) the lowest volume at which a visible auditory evoked potential (AEP) could be detected during NREM sleep. Because hearing sensitivity during sleep does not map directly onto wake hearing thresholds^27^, inter-individual differences in the required stimulation volume are expected even in a sample of young and healthy participants. This variability reflects meaningful differences in auditory responsiveness during sleep across participants, and we consider this a methodological strength, as it helps ensure the stimuli elicit a brain response without disrupting sleep. However, as already acknowledged in a previous publication from our laboratory^5^, the use of comfortable over-ear headphones in the sleep headband can lead to varying levels of pressure on the ears depending on the sleeping position. This may have affected the perceived volume and, therefore, the volume to which the stimulation was adapted during the adaptation night (as well as the magnitude of evoked responses in the EEG). We have integrated this aspect in the *Limitations* (p.27):

“Additionally, the headband included over-ear headphones for participant comfort, which bear the risk of applying varying levels of pressure to the ears depending on the sleeping position. This may have introduced additional variability in the perceived sound pressure level and the magnitude of stimulus-evoked responses.”

8. *Was auditory stimulation stopped when arousals occurred, or was it continuously presented? If it was not paused, I expect that it might have made it more difficult for participants to return to sleep after being awakened.*

Thank you for raising this important point. We agree that this mechanism for avoiding stimulation during arousals is a relevant factor that required clearer explanation. While this aspect was described in the original study^28^, we had not previously emphasized it in our Methods section. We have now explicitly added details on arousal detection and the stimulation-triggering criteria to the revised *Methods* section (p.9-10). It now reads:

“The algorithm distinguishes NREM (stages N2 and N3) from non-NREM sleep (wake, N1, and REM sleep) in real time based on elevated power in the low delta (2–4 Hz) and high delta (3–5 Hz), and low power in the high beta (20–30 Hz) band in the preceding 80 s of the EEG signal^28^. […]. Notably, the algorithm prevents stimulation immediately after arousals by detecting short-term (within the past second) increases in beta power, which are indicative of alertness or movement^29^.”

9. *If I understand correctly, all participants underwent the same training protocol across two nights. If so, sleep-related effects might have been more pronounced on the first night compared to the second. I wonder how this study design may have influenced training and sleep effects. Were performance measures analyzed separately for each night, or were the two nights combined?*

Performance measures were indeed analyzed separately for each night, as the short and long interstimulus-interval protocols (the two conditions) were applied on separate nights. You are correct that the order of conditions across the two intervention nights may influence the observed effects. To account for this, we employed a balanced cross-over design, with the order of conditions (previously termed “period”, now renamed to “night”, for clarity) randomized across participants. We had previously described this in the *Methods* section (p.10):

“During intervention nights, two up-PTAS protocols were administered in random order: the short ISI protocol and the long ISI protocol. Both protocols shared the same stimulation period, an ON-OFF block design, and a constant stimulation volume as derived from the adaptation phase but differed systematically in the interstimulus intervals.”

To minimize carryover learning effects the finger-tapping sequences were never reused within participants and were randomized across conditions while being matched for difficulty (Methods, p.8).

Finally, our statistical models explicitly controlled for potential order effects, which were previously detailed in the *Results* and which we have now moved to the *Supplemental Material* for brevity. In the *Results* (p.18), we now state the outcomes of these control analyses more clearly:

“Control analyses confirmed that these effects were unlikely to be driven by order effects or the distribution of sleep stages (**Supplemental Analyses** and **Tables S4-S5**)”.

10. *In the Abstract, the authors state: "As K-complexes may also be involved in cardiovascular function and brain waste clearance, the proposed protocol optimization may have effects beyond memory enhancement." This statement should be toned down, as the relationship between K-complexes and brain waste clearance is still unclear.*

Thank you for this thoughtful comment. In response, we have removed the sentence from the *Abstract* to avoid overstating the current evidence. However, as the potential link between K-complexes and brain waste clearance remains an interesting and emerging topic, we have retained the discussion of this point in the *Discussion*, where it is more appropriately addressed with the necessary nuance and context.

11. *Please clarify how the algorithm classifies EEG data into NREM and REM sleep based only on delta, high delta, and high beta bands. Does it not incorporate EMG or EOG recordings? How does it detect wakefulness or movement?*

Thank you for this important question. While the device records EOG and EMG signals, the stimulation algorithm distinguishes NREM sleep from non-NREM sleep solely based on the EEG signal from the frontal electrode. As you noted, this classification depends on delta, high delta and high beta bands. As shown in the original publication^28^, increases in beta power — associated with alertness, artifacts, and arousals^29^—, are sufficient for the algorithm to avoid stimulating outside of stable NREM sleep. Thus, although the algorithm does not use EMG or EOG data, it effectively minimizes stimulation during periods of wakefulness or movement by relying on these EEG-based spectral features. To clarify this point, we have expanded the *Methods* section (p.9-10) to include a more detailed description of the stimulation algorithm (as also addressed in response to an earlier comment).

**References**

1.	Fischer S, Hallschmid M, Elsner AL, Born J. Sleep forms memory for finger skills. *Proceedings of the National Academy of Sciences*. 2002;99(18):11987-11991. doi:10.1073/pnas.182178199

2.	Fattinger S, De Beukelaar TT, Ruddy KL, et al. Deep sleep maintains learning efficiency of the human brain. *Nat Commun*. 2017;8(May):1-13. doi:10.1038/ncomms15405

3.	Walker MP, Brakefield T, Morgan A, Hobson JA, Stickgold R. Practice with Sleep Makes Perfect: Sleep-Dependent Motor Skill Learning. *Neuron*. 2002;35:205-211. doi:10.1016/S0896-6273(02)00746-8

4.	Rasch B, Pommer J, Diekelmann S, Born J. Pharmacological REM sleep suppression paradoxically improves rather than impairs skill memory. *Nat Neurosci*. 2009;12(4):396-397. doi:10.1038/nn.2206

5.	Leach S, Krugliakova E, Sousouri G, et al. Acoustically evoked K-complexes together with sleep spindles boost verbal declarative memory consolidation in healthy adults. *Sci Rep*. 2024;14(1). doi:10.1038/s41598-024-67701-7

6.	Wunderlin M, Züst MA, Hertenstein E, et al. Modulating overnight memory consolidation by acoustic stimulation during slow-wave sleep: A systematic review and meta-analysis. *Sleep*. 2021;44(7). doi:10.1093/sleep/zsaa296

7.	Leminen MM, Virkkala J, Saure E, et al. Enhanced memory consolidation via automatic sound stimulation during non-REM sleep. *Sleep*. 2017;40(3). doi:10.1093/sleep/zsx003

8.	Schneider J, Lewis PA, Koester D, Born J, Ngo HV V. Susceptibility to auditory closed-loop stimulation of sleep slow oscillations changes with age. *Sleep*. 2020;43(12). doi:10.1093/sleep/zsaa111

9.	Bellesi M, Riedner BA, Garcia-Molina GN, Cirelli C, Tononi G. Enhancement of sleep slow waves: Underlying mechanisms and practical consequences. *Front Syst Neurosci*. 2014;8(208):1-17. doi:10.3389/fnsys.2014.00208

10.	Esfahani MJ, Farboud S, Ngo HV V, et al. Closed-loop auditory stimulation of sleep slow oscillations: Basic principles and best practices. *Neurosci Biobehav Rev*. 2023;(153). doi:10.1016/j.neubiorev.2023.105379

11.	Lustenberger C, Ferster ML, Huwiler S, et al. Auditory deep sleep stimulation in older adults at home: a randomized crossover trial. *Communications Medicine*. 2022;2(1). doi:10.1038/s43856-022-00096-6

12.	Ngo HV V., Martinetz T, Born J, Mölle M. Auditory closed-loop stimulation of the sleep slow oscillation enhances memory. *Neuron*. 2013;78(3):545-553. doi:10.1016/j.neuron.2013.03.006

13.	Cox R, Korjoukov I, De Boer M, Talamini LM. Sound Asleep: Processing and Retention of Slow Oscillation Phase-Targeted Stimuli. *PLoS One*. 2014;9(7):101567. doi:10.1371/journal.pone.0101567

14.	Navarrete M, Osorio-Forero A, Gómez A, et al. Response of sleep slow oscillations to acoustic stimulation is evidenced by distinctive synchronization processes. *Sleep*. 2023;46(6). doi:10.1093/sleep/zsad110

15.	Huwiler S, Carro Dominguez M, Huwyler S, et al. Effects of auditory sleep modulation approaches on brain oscillatory and cardiovascular dynamics. *Sleep*. 2022;45(9). doi:10.1093/sleep/zsac155

16.	Schönauer M, Geisler T, Gais S. Strengthening procedural memories by reactivation in sleep. *J Cogn Neurosci*. 2014;26(1):143-153. doi:10.1162/jocn_a_00471

17.	Ngo HV V., Miedema A, Faude I, Martinetz T, Mölle M, Born J. Driving sleep slow oscillations by auditory closed-loop stimulation—A self-limiting process. *Journal of Neuroscience*. 2015;35(17):6630-6638. doi:10.1523/JNEUROSCI.3133-14.2015

18.	de Beukelaar TT, Woolley DG, Wenderoth N. Gone for 60 seconds: Reactivation length determines motor memory degradation during reconsolidation. *Cortex*. 2014;59:138-145. doi:10.1016/j.cortex.2014.07.008

19.	Kuriyama K, Stickgold R, Walker MP. Sleep-dependent learning and motor-skill complexity. *Learning and Memory*. 2004;11(6):705-713. doi:10.1101/lm.76304

20.	Fernández-Varela I, Alvarez-Estevez D, Hernández-Pereira E, Moret-Bonillo V. A simple and robust method for the automatic scoring of EEG arousals in polysomnographic recordings. *Comput Biol Med*. 2017;87:77-86. doi:10.1016/j.compbiomed.2017.05.011

21.	Alvarez-Estevez D, Fernández-Varela I. Large-scale validation of an automatic EEG arousal detection algorithm using different heterogeneous databases. *Sleep Med*. 2019;57:6-14. doi:10.1016/j.sleep.2019.01.025

22.	Rasch B, Born J. About Sleep’s Role in Memory. *Physiologial Reviews*. 2013;93:681-766. doi:10.1152/physrev.00032.2012

23.	Chauvette S, Seigneur J, Timofeev I. Sleep Oscillations in the Thalamocortical System Induce Long-Term Neuronal Plasticity. *Neuron*. 2012;75(6):1105-1113. doi:10.1016/j.neuron.2012.08.034

24.	Firth H. Habituation During Sleep. *Psychophysiology*. 1973;10(1):43-51. doi:10.1111/j.1469-8986.1973.tb01081.x

25.	Bastien C, Campbell K. Effects of rate of tone‐pip stimulation on the evoked K‐Complex. *J Sleep Res*. 1994;3(2):65-72. doi:10.1111/j.1365-2869.1994.tb00109.x

26.	Roth M, Shaw J, Green J. The form, voltage distribution and physiological significance of the K-complex. *Electroencephalogr Clin Neurophysiol*. 1956;8(3):385-402.

27.	Rosenthal L, Bishop C, Helmus T, Krstevska S, Roehrs T, Roth T. *Arousals During Sleep and Sleep Disruption Auditory Awakening Thresholds in Sleepy and Alert Individuals*. Vol 19.; 1996. https://academic.oup.com/sleep/article/19/4/290/2749817.

28.	Ferster ML, Lustenberger C, Karlen W. Configurable Mobile System for Autonomous High-Quality Sleep Monitoring and Closed-Loop Acoustic Stimulation. *IEEE Sens Lett*. 2019;3(5). doi:10.1109/LSENS.2019.2914425

29.	Li G, Lee BL, Chung WY. Smartwatch-Based Wearable EEG System for Driver Drowsiness Detection. *IEEE Sens J*. 2015;15(12):7169-7180. doi:10.1109/JSEN.2015.2473679
